# METTL16 suppressed the proliferation and cisplatin-chemoresistance of bladder cancer by degrading PMEPA1 mRNA in a m6A manner through autophagy pathway

**DOI:** 10.7150/ijbs.86719

**Published:** 2024-02-04

**Authors:** Hao Yu, Juntao Zhuang, Zijian Zhou, Qiang Song, Jiancheng Lv, Xiao Yang, Haiwei Yang, Qiang Lu

**Affiliations:** 1 Department of Urology, The First Affiliated Hospital of Nanjing Medical University, Nanjing 210029, China.; 2 Laboratory of Urology and Andrology, Jiangsu Clinical Medicine Research Institution, Nanjing 210029, China.

**Keywords:** METTL16, bladder cancer, PMEPA1, autophagy, HIF-2α

## Abstract

N6-methyladenosine (m6A) is important in the physiological processes of many species. Methyltransferase-like 16 (METTL16) is a novel discovered m6A methylase, regulating various tumors in an m6A-dependent manner. However, its function in bladder cancer (BLCA) remains largely unclear. In the present study, we found that low expression of METTL16 predicted poor survival in BLCA patients. METTL16 inhibited the proliferation and cisplatin-resistance function of bladder cancer cells *in vitro* and *in vivo*. In addition, METTL16 reduced the mRNA stability of prostate transmembrane protein androgen induced-1 (PMEPA1) via binding to its m6A site in the 3'-UTR, thereby inhibited the proliferation of bladder cancer cells and increased the sensitivity of cisplatin through PMEPA1-mediated autophagy pathway. Finally, we found that hypoxia-inducible factor 2α (HIF-2α) exerted its tumor-promoting effect by binding the METTL16 promoter region to repress its transcription. Taken together, High expression of METTL16 predicted better survival in BLCA. METTL16 significantly inhibited bladder cancer cell proliferation and sensitized bladder cancer cells to cisplatin via HIF-2α-METTL16-PMEPA1-autophagy axis in a m6A manner. These findings might provide fresh insights into BLCA therapy.

## Introduction

Bladder cancer is the tenth most common malignancy in the world, with about 570,000 new cases and 210,000 deaths in 2020^1^. For several years, cisplatin-based chemotherapy has been the most combination treatment for bladder cancer, significantly improving 5-year overall survival in chemotherapy-sensitive patients. However, the overall response rate to chemotherapy is only around 20-40%^2^. Many studies have shown that tumor epigenetics and tumor microenvironment may play an important role in tumor proliferation and drug resistance^3^. Therefore, finding new therapeutic targets and improving the effectiveness of existing drugs are of great meaning.

As the most abundant post-transcriptional modification in RNA of eukaryote^4^, N6-methyladenosine (m6A) regulates RNA splicing^5^-^8^, stability^9^-^11^ and translation^12^-^14^ by influencing tRNAs, rRNAs, long noncoding RNAs, circular RNAs, snRNAs and mRNA methylation. Several important biological processes are altered via m6A “writers” (METTL3-METTL14 complex, METTL16), “erasers” (FTO, ALKBH5) and “readers” (YTHDC family and IGF2BP family) proteins, functioning as catalyzing, removing, or recognizing m6A-modified sites respectively^15^. Multiple studies have reported that m6A regulatory proteins fulfill important roles in the initiation and progression of bladder cancer^16^-^20^. In our laboratory, Han *et al.*^21^ reported that METTL3 promoted tumor proliferation of bladder cancer by accelerating pri-miR221/222 maturation in an m6A-dependent manner. And Yu *et al.* 22 found ALKBH5 inhibited cell proliferation and sensitized bladder cancer cells to cisplatin by m6A-CK2a-mediated glycolysis. Other researchers also showed that m6A regulatory protein is closely related to bladder cancer. Tao *et al.* 23 reported that FTO facilitated the tumorigenesis of bladder cancer through regulating the MALAT/miR-384/MAL2 axis in m6A RNA modification manner. Xie *et al.* 24 confirmed that METTL3/YTHDF2 m6A axis promoted tumorigenesis by degrading SETD7 and KLF4 mRNAs in bladder cancer. Gu *et al.* 25 found that METTL14 inhibited bladder TIC self-renewal and bladder tumorigenesis through N6-methyladenosine of NOTCH1. All the above research indicated that m6A methylation may play an important role in bladder cancer.

The m6A methyltransferase complex, composed of METTL3 and METTL14, has been thought to be the main m6A writer^26^, ^27^. However, depletion of METTL3 and METTL14 is known to cause about 60% decrease of m6A modification in some cell types, with large portions of m6A marks in cellular do not map well with the binding sites of METTL3-METTL14^28^. These observations imply the existence of additional m6A methyltransferase. Recently, METTL16 (methyltransferase-like 16, also known as METT10D), which belongs to the methyltransferase like (METTL) proteins family, is identified as a novel “writer” that could drive methylation independently of the methyltransferase complex, inducing initiation or progression in hepatocellular cancer^29^, pancreatic ductal adenocarcinoma^30^, leukemia^31^, breast cancer^32^, gastric cancer^33^, cervical cancer^34^ and so on^35^-^37^. In addition, Zheng* et al.* 38 predicted that METTL16 may be a good prognostic factor for bladder cancer through bioinformatics analysis. However, the mechanism and role of METTL16 in bladder cancer have yet to be elucidated.

The level/activity of m6A writers, erasers, and readers can be regulated by hypoxia^39^. Hypoxia is the common condition occurring in the tumor microenvironment (TME) of solid tumors^40^. In fact, hypoxia is a known poor prognosis marker, driving therapy resistance^41^, ^42^, angiogenesis^43^, ^44^, metastasis^45^, ^46^ and tumor progression^47^, ^48^. The backbone in this adaptive response is the activation of the hypoxia inducible factors (HIFs), HIF-1 and HIF-2^49^. HIFs' activation in response to tumor hypoxia can drive tumor adaptation and development^50^. Hypoxia-induced expression is due to direct binding of HIFs to hypoxia response elements (HREs) in target genes' promoter region, which contain the core HIF-binding site sequence “(A/G) CGTG”. HIFs had been reported to affect m6A level in tumor via interfering with transcription of m6A-related genes by binding to the HREs of promoter region 51-54. However, the correlation of HIF-2α with m6A related genes has not been exploited in the bladder cancer.

Autophagy is the major intracellular degradation system through which cytoplasmic materials are delivered to and degraded in the lysosome^55^. The role of autophagy in tumors is currently controversial. But the general consensus is that when tumors progress to advanced stages and face a hostile environment, autophagy acts as a dynamic degradation and recycling system that contributes to the survival of established tumors^56^, ^57^. It has been reported that bladder cancer also exhibits high basal levels of autophagic activities^58^, ^59^. Currently, some evidence have been proved that autophagy is a protective mechanism to promote the proliferation^60^, ^61^ and against cisplatin-based chemotherapy^62^-^64^ in cancer. In addition, Li* et al.*^65^ reported that autophagy induced by starvation promotes the progression of bladder cancer cell T24 and UMUC3 by LDHA mediated metabolic reprogramming. Thomas *et al.*^66^ demonstrated the autophagy is a protective factor of bladder cancer in chemotherapy to cisplatin and miR-30a-3p could overcome it. Therefore, targeting autophagy in human bladder cancer seems to be a reasonable therapeutic approach.

In the present study, we sought to investigate the roles and associated mechanisms of METTL16 in bladder cancer. These experiments revealed the following: (1) METTL16 expression was downregulated in bladder cancer tissues, and low expression levels of METTL16 was associated with a poor prognosis; (2) METTL16 inhibited the proliferation of bladder cancer cells and increased the sensitivity to cisplatin *in vitro* and *in vivo*; (3) METTL16 played a suppressive role in bladder cancer by reducing the stability of PMEPA1 mRNA in an m6A-dependent manner, and affecting the autophagy pathway; (4) Under hypoxia, HIF-2α negatively regulated METTL16 expression, and inhibited its transcription by binding to the METTL16 promoter region. To our knowledge, this is the first comprehensive study to verify that METTL16 may regulate the progression of bladder cancer in an m6A-dependent manner and it is also the first to explore the possible regulatory mechanisms upstream of METTL16 in solid tumors. The results obtained in our experiments may provide new insights for the development of new therapies for bladder cancer.

## Results

### METTL16 was Significantly Downregulated in Human Bladder Cancer Tissues and Associated with Bladder Cancer Patient Prognosis

**Figure 1:**
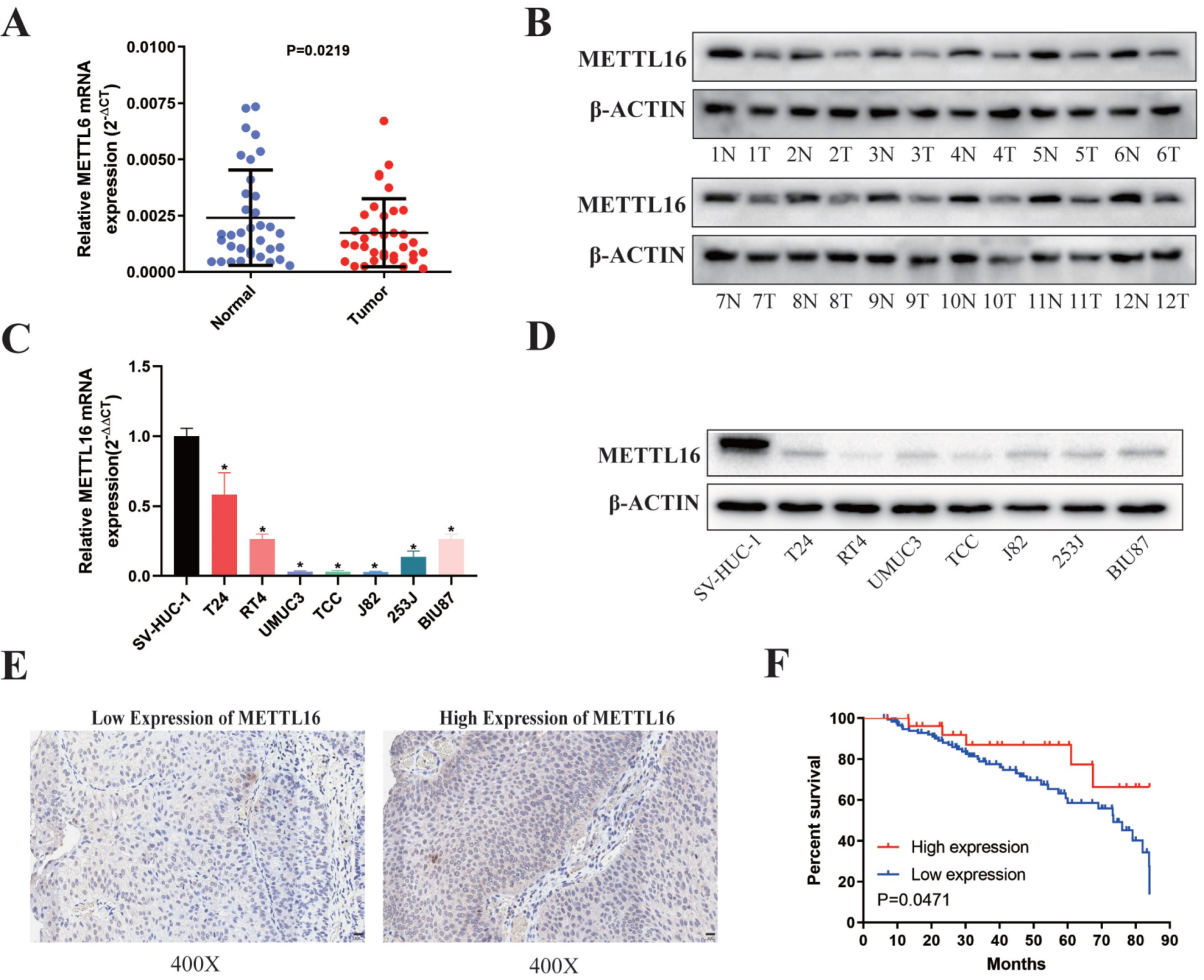
** METTL16 was downregulated in bladder cancer tissues and cell lines and associated with patient prognosis. (A)** Relative expression of METTL16 mRNA in the 35 pairs of bladder cancer tissues and matched adjacent normal tissues quantified by qRT-PCR. METTL16 was down-regulated in bladder cancer tissues compared with that in adjacent normal tissues (*P*< 0.05). **(B)** The expression of METTL16 protein level in 12 pairs of bladder cancer tissues (T) and adjacent normal tissues (N) by western blot. **(C and D)** Relative expression of METTL16 in bladder cancer cell lines and normal bladder epithelial cell line SV-HUC-1 by qRT-PCR and western blot. Data represent the mean ± SD from three independent experiments, * P < 0.05. **(E)** IHC analysis of METTL16 in bladder cancer tissue at 400X magnification. Scale bars indicated 20 μm.** (F)** Kaplan-Meier survival curves of overall survival in 151 bladder cancer patients based on METTL16 by IHC staining. The log-rank test was used to compare differences between two groups (P = 0.047).

First, we analyzed 35 paired bladder cancer tissues and found that the RNA (Figure 1A) and protein (Figure 1B) expression of METTL16 in bladder cancer tissues were significantly downregulated compared with paired adjacent tissues. METTL16 was also downregulated in seven bladder cancer cell lines compared with SVHUC-1 (human ureteral epithelial immortalized cell line, as the normal urothelial cell line) (Figure 1C,1D). Moreover, we analyzed tissue microarray (TMA) to explore the relationship between METTL16 expression and clinicopathological of patients by using immunohistochemistry (IHC) (Figure 1E). We found that METTL16 expression was related to TNM stage (Table 1). Notably, we observed lower TNM stage in patients with tumors expressing higher METTL16 levels, as well as better prognosis and overall survival (Figure 1F). The same results were obtained from BLCA data set TCGA database in UALCAN (Figure S1A,1B) and The Human Protein Atlas (HPA) database (Figure S1D,1E). Therefore, we speculated that METTL16 acted as a suppressor in bladder cancer.

### METTL16 Inhibited Bladder Cancer Cell Proliferation and Cisplatin- Resistance *in vivo* and *in vitro*

**Figure 2:**
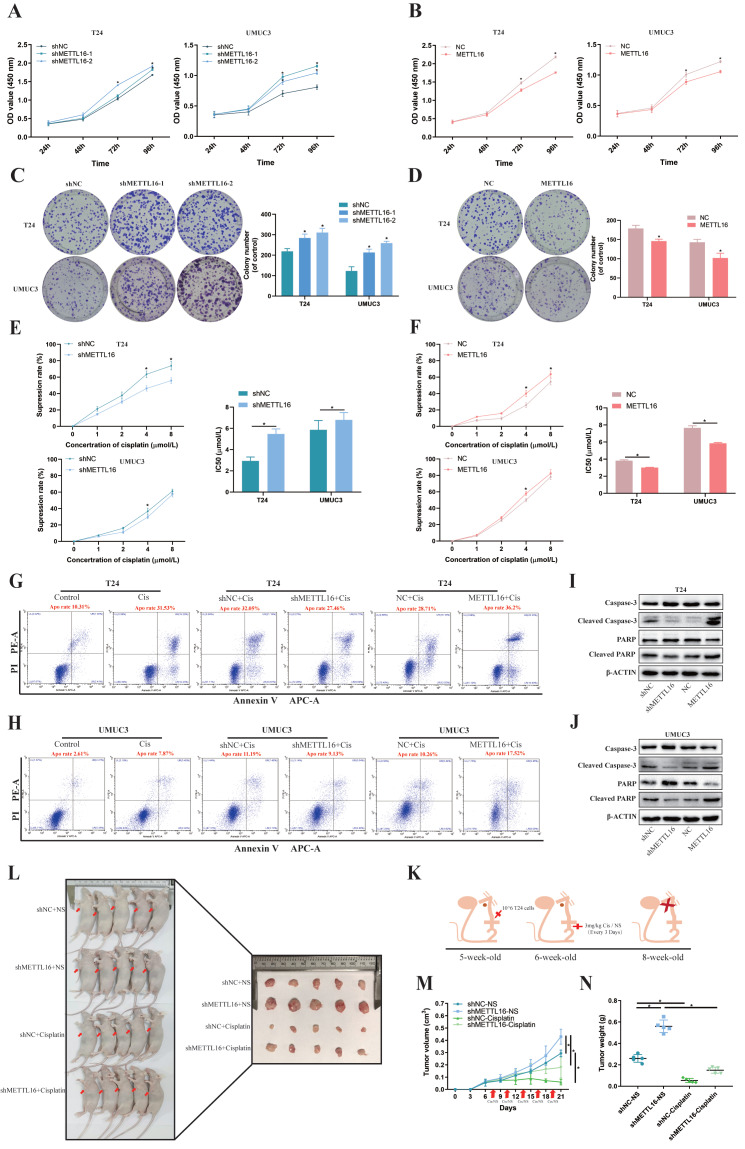
** METTL16 inhibited bladder cancer cell proliferation and cisplatin- resistance *in vivo* and *in vitro.* (A and B)** Cell proliferation assessed by CCK8 assays. Knockdown of METTL16 promoted cell proliferation in T24 and UMUC3 cells. Overexpression of METTL16 inhibited cell proliferation in T24 and UMUC3 cells. Data represent the mean ± SD from three independent experiments, *P < 0.05. **(C and D)** Colony formation assay showed that knockdown of METTL16 significantly increased the cloning number of T24 and UMUC3 cells compared with control group, while METTL16 overexpression significantly decreased the cloning number of T24 and UMUC3. Data represent the mean ± SD from three independent experiments, *P < 0.05. **(E)** Knockdown of METTL16 expression decreased the suppression rate of cisplatin and increased IC50 to cisplatin in T24 and UMUC3 cells by CCK8. Data represent the mean ± SD from three independent experiments, *P < 0.05. **(F)** Overexpression of METTL16 increased cisplatin-induce apoptosis rate and decreased IC50 to cisplatin in T24 and UMUC3 cells by CCK8. Data represent the mean ± SD from three independent experiments, *P < 0.05.** (G and H)** Flow cytometry assay showed the percentage of apoptotic cells was increased after treated with cisplatin for 36 hours. Knockdown of METTL16 expression decreased the rate of cisplatin-induced apoptosis compared with control cells in T24 and UMUC3 cells, whereas overexpression METTL16 increased the rate of cisplatin-induced apoptosis. **(I and J)** After treated with cisplatin for 36 hours, western blot analysis of apoptosis-related proteins caspase3, PARP, Cleaved -caspase3, Cleaved-PARP and β-Actin in T24 and UMUC3 cells with METTL16 knockdown or overexpression. The level of caspase3, PARP was increased and Cleaved -caspase3, Cleaved-PARP were decreased in knockdown group compared with NC control group, whereas overexpressed METTL16 elicited the opposite effect in T24 and UMUC3.** (K)** Subcutaneous xenograft tumor model with METTL16 knockdown (shMETTL16) or control cells (shNC). Cisplatin or normal saline was injected intraperitoneally starting from day 7 of tumor inoculation. The red arrows indicated the location of the tumor.** (L)** Establishment and experimental design of subcutaneous xenograft tumor model. **(M)** Tumor volume was measured every 3 days from transplanting. Data represent the mean ±SD, *P<0.05. **(N)** Tumor weight was measured after executing the mouse. Data represent the mean ±SD, *P<0.05.

To verify the function of METTL16 *in vitro*, T24 and UMUC3 cells were stably transfected with knockdown lentivirus, overexpression lentivirus, or control lentivirus. The expression levels of METTL16 were confirmed by qRT-PCR analysis (Figure S2A,2C) and western blotting (Figure S2B,2D). Cell counting kit-8 (CCK-8) assays and colony formation indicated that METTL16 knockdown significantly increased cell proliferation (Figure 2A,2C), while METTL16 overexpression decreased cell proliferation (Figure 2B,2D). In addition, based on TCGA database and Drug Sensitivity in Cancer (GDSC) database analysis, results showed a positive correlation between METTL16 levels and cisplatin chemotherapy sensitivity in bladder tumors (Figure S1C). Therefore, we used CCK-8 and flow cytometer to determine the effect of METTL16 on cisplatin sensitivity. The result of CCK-8 showed that with the increase of cisplatin concentration, the cell activity was significantly inhibited. Compared with the negative control group (shNC), METTL16 knockdown significantly reduced the inhibition rate of cisplatin on T24 and UMUC3 cells (Figure 2E). The half-maximal inhibitory concentration (IC50) results showed that knockdown of METTL16 led to increased IC50 value and decreased cisplatin sensitivity (Figure 2E), while overexpression of METTL16 elicited the opposite effect (Figure 2F). In addition, we also found that the apoptosis rate of T24 increased significantly after cisplatin-treatment for 36 h. METTL16 knockdown decreased cisplatin-induced apoptosis, while METTL16 overexpression increased the apoptosis (Figure 2G). The same results were found in the UMUC3 cell line (Figure 2H). Western blotting results showed that after 36 h of cisplatin treatment, the level of apoptosis-related proteins caspase3, PARP was increased and Cleaved -caspase3, Cleaved-PARP were decreased in knockdown group compared with NC control group, whereas overexpressed METTL16 elicited the opposite effect in T24 (Figure 2I) and UMUC3 (Figure 2J). *In vivo*, T24 cells transfected with knocking down of METTL16 or the control group were injected subcutaneously into nude mice (Figure 2K). The results showed that METTL16 knockdown increased the size and weight of bladder cancer tumors in mice (Figure 2L). Compared with the saline group, the tumor volume (Figure 2M) and weight (Figure 2N) in the cisplatin treatment group were reduced. Although the tumor volume and weight in the METTL16 knockdown group were slightly larger than those in the NC group, the changes were far less significant than those in the saline group. It was further confirmed that knockdown of METTL16 could promote proliferation and reduce the sensitivity to cisplatin chemotherapy of bladder tumor cell T24 to a certain extent *in vivo*. However, cell cycle analysis revealed that METTL16 knockdown did not cause obvious cycle changes in bladder cancer cells (Figure S3A). This indicated that METTL16 may regulate the biological function of bladder cancer in a cell cycle independent manner. Thus, our data suggested that METTL16 can inhibit the proliferation of bladder cancer and improve the apoptosis rate and cisplatin sensitivity in bladder cancer cells.

### METTL16 was Negatively Correlated with PMEPA1 Expression in Bladder Cancer Tissues and Cell Lines

**Figure 3:**
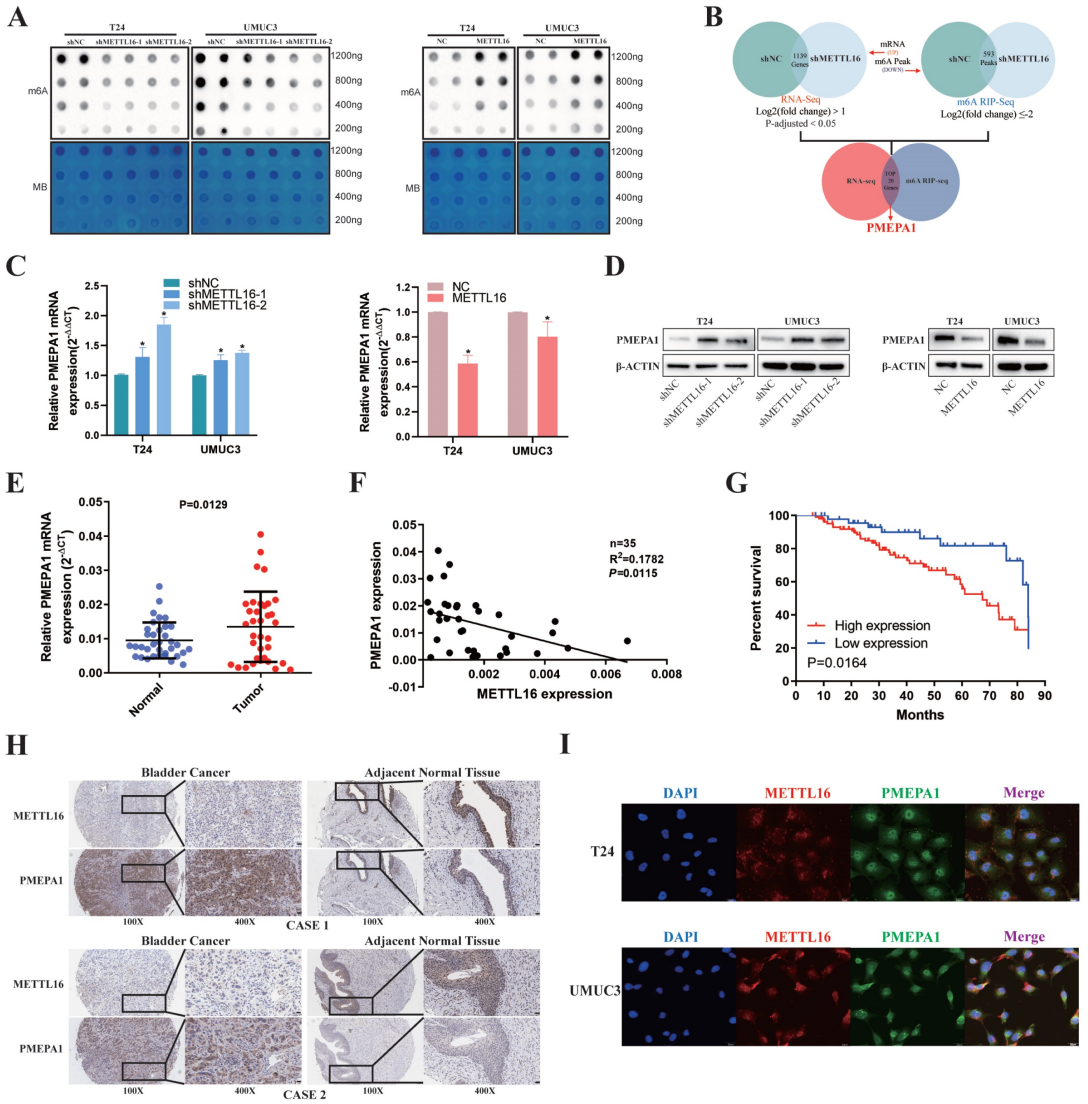
** METTL16 was negatively correlated with PMEPA1 expression in bladder cancer tissues and cell lines. (A)** M6A dot blot assays of T24 and UMUC3 cells with knockdown or overexpression of METTL16. Methylene blue (MB) stain as loading control. The methylation of RNA decreased after METTL16 knockdown, while increased after METTL16 overexpression. **(B)** Schematic of RNA-seq and MeRIP-seq (design and result). **(C and D)** QRT-PCR and western blot analysis of PMEPA1 in T24 and UMUC3 cells with METTL16 knockdown or overexpression. Data represent the mean ± SD from three independent experiments, *P < 0.05. **(E)** PMEPA1 was higher expression in bladder cancer tissues than in adjacent normal tissues by qRT-PCR (n=35, *P*<0.05). **(F)** A negative correlation between the expression of PMEPA1 and METTL16 was showed in bladder cancer tissues by qRT-PCR. Data represent the mean ± SD from three independent experiments, *P < 0.05.** (G)** Kaplan-Meier survival curves of overall survival in 151 bladder cancer patients based on PMEPA1 by IHC staining. The log-rank test was used to compare differences between two groups (P = 0.0164). **(H)** IHC analysis of METTL16 and PMEPA1 in bladder cancer tissue or matched adjacent normal tissues at 100X (left) or 400X (right) magnification and scale bars indicated 100 μm (100X), 20 μm (400X). Two cases were presented.** (I)** Immunofluorescence staining of T24 and UMUC3 cells revealed that METTL16 and PMEPA1 was widely distributed in the nucleus and cytoplasm. Scale bars indicated 20 μm.

First, we used m6A dot blot to explore the role of METTL16 in regulating m6A modification in bladder cancer. Knockdown of METTL16 resulted in a decrease of m6A levels in T24 and UMUC3 cells, while METTL16 overexpression increased m6A levels (Figure 3A). To identify the exact mechanism supporting the observed METTL16 phenotype, we sequenced T24 cells with lentivirus knockdown of METTL16 and virus control using a combination of RNA-seq and MeRIP-seq. After knockdown of METTL16, the abundance of 593 differential m6A peaks was decreased (log2(fold change) ≤-2). As we attached importance to the potential m6A oncogenic targets whose methylation and expression levels were regulated by METTL16, we focus on the transcripts whose expression were increased by METTL16 knockdown in RNA-seq at the same time. After knockdown of METTL16, 1139 genes' transcript was decreased (log2(fold change) >1, P-adjusted<0.05). Therefore, only those with low m6A peak and elevated transcript levels after knocking down METTL16 were selected for the following study (Figure 3B). We selected the top 20 genes (SLC16A9, SLC25A27, ARID5B, ARSI, RP1, HMGN5, NCKAP5, PCDH7, PRRT2, LINC01910, SNHG18, PCDH18, ABCC3, RHOBTB1, GLIS3, TENM4, ELFN2, CAMKK1, PMEPA1, THRB) and comprehensively analyzed these genes using BLCA data set TCGA database. We found only PMEPA1 gene were noticeably over-expressed in tumor tissues (Figure S4A) and a poor prognosis (Figure S4B). Notably, we observed higher TNM stage and nodal metastasis in patients with higher PMEPA1 expression (Figure S4C,4D). Subsequently, we found the mRNA (Figure 3C) and protein level (Figure 3D) of PMEPA1 were reversely regulated by METTL16 in T24 and UMUC3 cells. Hence, we used RNA (Figure 3E) and TMA (Figure 3H) of our own patients and found that PMEPA1 was remarkably higher expressed in tumor tissues than in adjacent tissues, while METTL16 was negatively correlated with PMEPA1 expression (Figure 3F, 3H, Figure S4E). Kaplan-Meier curves from TMA (Figure 3G) and HPA database (Figure S4F) also indicated that high expression of PMEPA1 led to poor prognosis. Furthermore, immunofluorescence results indicated that the expression of METTL16 and PMEPA1 occurred in both nucleus and cytoplasm in T24 and UMUC3 cells (Figure 3I). IHC of mouse subcutaneous tumors also demonstrated a negative correlation between METTL16 and PMEPA1 expression (Figure S4G). According to TMA, the clinical features of METTL16 were also negative correlation of PMEPA1 (Table 1). In addition, PMEPA1 was also shown to promote bladder cancer in TMA (Table S5). Combined with the bioinformatics prediction and the above results, PMEPA1 was likely to be the direct downstream target of METTL16.

### METTL16 Reduced the Stability of the PMEPA1 mRNA in an m6A Dependent Manner

**Figure 4:**
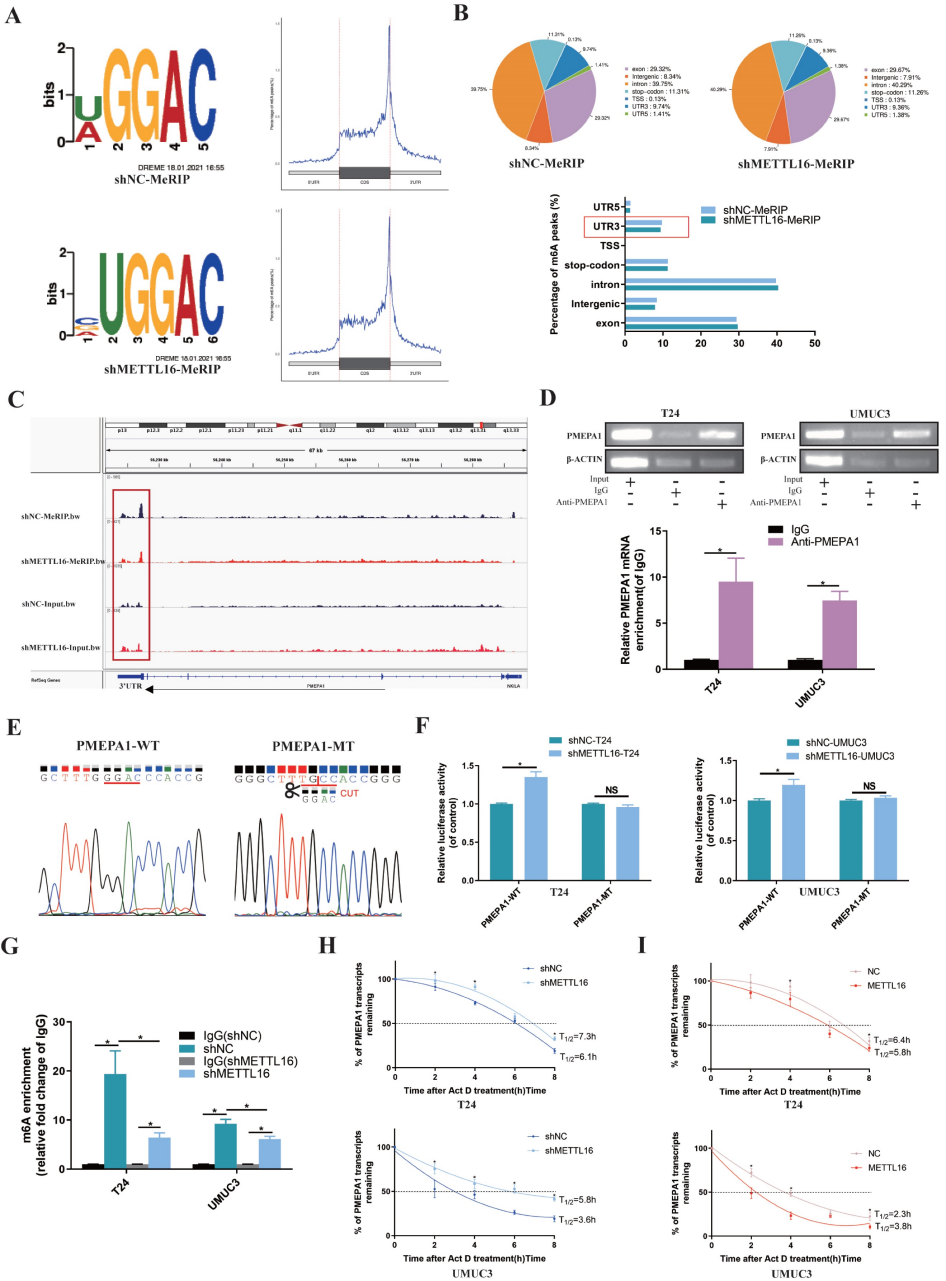
** METTL16 affected the stability of the PMEPA1 mRNA in an m6A dependent manner. (A)** Sequence motifs of shNC group and shMETTL16 group in m6A peaks identified by MeRIP-seq result.** (B)** Pie chart depicting the fraction of m6A peaks in 7 transcript segments in shNC group and shMETTL16 group. Knocking down METTL16 significantly reduced m6A PEAK in the 3'-UTR.** (C)** Integrative genomics viewer (IGV) plots of m6A peaks at PMEPA1 mRNAs. The m6A peak of PMEPA1 in the METTL16 knockdown group was apparently reduced in the 3'-UTR, compared with the control group, whereas input group elicited the opposite effect.** (D)** RIP assay demonstrated that anti‑METTL16 antibody was able to significantly enrich the level of PMEPA1 mRNA compared with the anti‑IgG antibody by qRT-PCR. β‑actin transcript was used as control.** (E)** The sequence of wild and deletion mutant Plenti-Utr-Luc reporter plasmids carrying the m6A sites (GGAC) or not of the 3'-UTR of PMEPA1.** (F)** The effect of knocking down METTL16 on a wild type Plenti-Utr-Luc vector or a deletion mutant Plenti-Utr-Luc vector was measured by luciferase reporter assays in T24 and UMUC3 cells.** (G)** The detection of PMEPA1 m6A modification level by immunoprecipitation of m6A modified mRNA in control or METTL16 knockdown cells followed by qRT-PCR. Data represent the mean ± SD from three independent experiments, *P < 0.05.** (H)** Knockdown of METTL16 expression increased the half-life of PMEPA1 transcript. Data represent the mean ± SD from three independent experiments, *P < 0.05. **(I)** Overexpression of METTL16 expression shortened the half-life of PMEPA1 transcript. Data represent the mean ± SD from three independent experiments, *P < 0.05.

Firstly, the results of MeRIP-seq demonstrated that consensus motif search identified “UGGAC” sequence (Figure 4A) of target gene. When METTL16 was downregulated, the overall Peak in the non-coding region was reduced and the most obvious decrease of m6A peaks was in 3'-UTR (3' untranslated regions) of target gene (Figure 4B). After analyzing the MeRIP-seq data by IGV (Itegrative Genomics Viewer) visualization software, the m6A peak of PMEPA1 in the METTL16 knockdown group was apparently reduced in the 3'-UTR, compared with the control group, whereas input group elicited the opposite effect (Figure 4C). Therefore, we speculated that METTL16 might bind to the m6A sites in the 3'-UTR of PMEPA1 mRNA. Subsequently, RIP assay showed that anti-METTL16 antibody significantly enriched PMEPA1 mRNA compared with anti-immunoglobulin G (IgG) antibody in T24 and UMUC3 cells (Figure 4D). These results indicated that METTL16 was able to bind to the PMEPA1 transcript. In addition, to investigate whether METTL16 can reduce PMEPA1 expression by binding to the m6A site of the 3'-UTR, wild and deletion mutant Plenti-Utr-Luc reporter plasmids carrying the m6A sites (GGAC) in the 3'-UTR region of PMEPA1 were constructed based on the sequencing results (Figure 4E). We transfected the plasmids into METTL16 knockdown T24 and UMUC3 cells and control cells. The dual-luciferase reporter assay results demonstrated that knock down METTL16 increased the luciferase activity of the wild type, but this phenomenon was restored in mutation group (Figure 4F). These data indicated that METTL16 can bind to the m6A site in the 3'-UTR region of PMEPA1. Finally, MeRIP assays showed that knockdown of METTL16 decreased the m6A levels of the PMEPA1 mRNAs in T24 and UMUC3 cells (Figure 4G). Large numbers of research results implied that m6A methylation occurring in the 3'-UTR will affect the mRNA stability of genes^16^, ^67^-^71^. Therefore, we used Actinomycin D (Act D) assay to deal with METTL16 knockdown or overexpressed cells and found that knockdown of METTL16 significantly prolonged the relative half-life of PMEPA1 transcripts (Figure 4H). On the contrary, overexpressed METTL16 shortened the half-life of PMEPA1 transcripts in T24 and UMUC3 cells (Figure 4I). It suggested that METTL16 reduced the stability of PMEPA1 mRNA. Taken together, all these results suggested that METTL16 reduced the stability of PMEPA1 mRNA in a m6A-dependent manner.

### METTL16 may Affect Bladder Cancer Proliferation and Cisplatin Resistant through Autophagy Pathway

**Figure 5:**
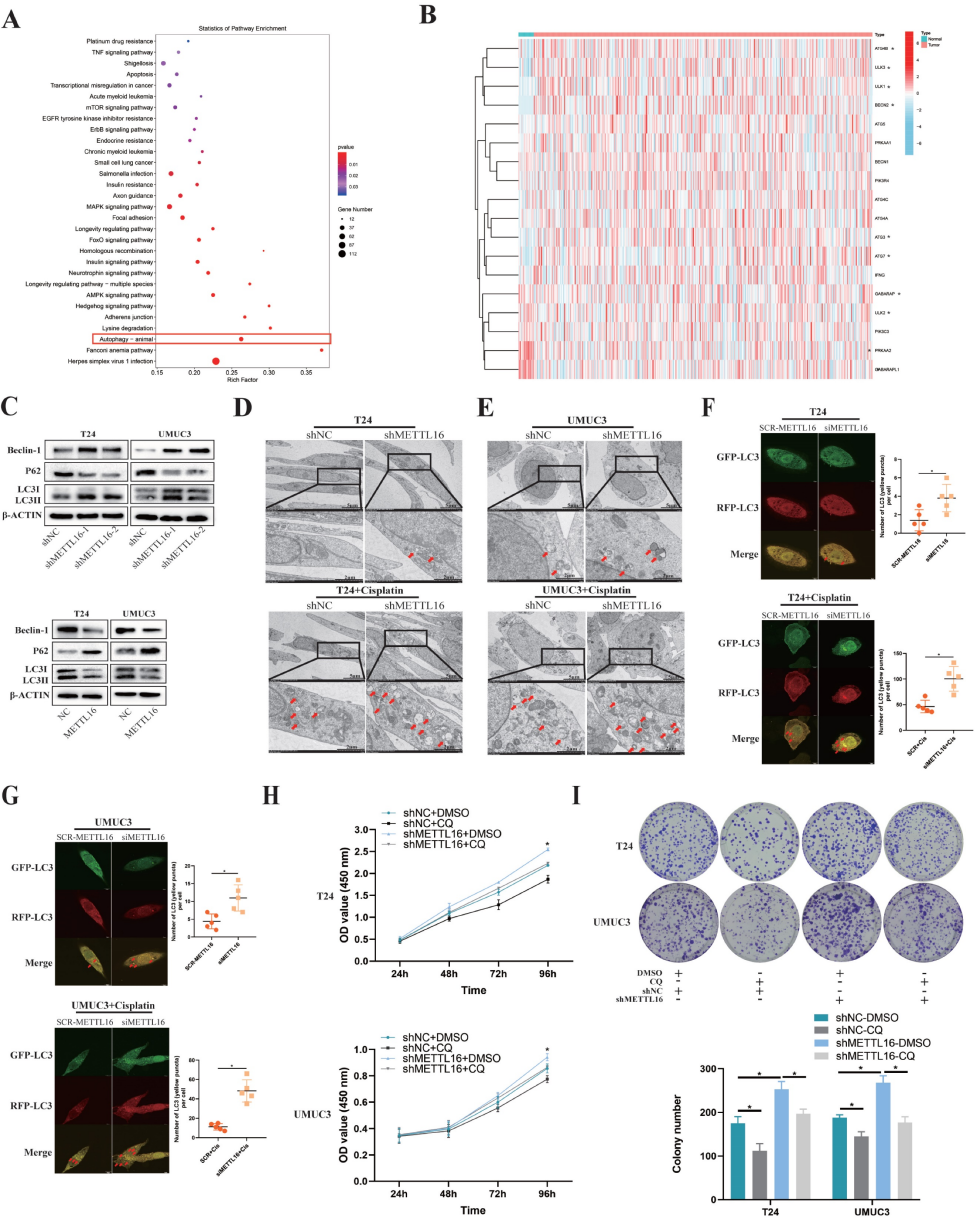
** METTL16 affected bladder cancer proliferation and cisplatin resistant via autophagy pathway. (A)** KEGG pathway analysis of differentially expressed genes from the sequence assay between shMETTL16 group and the control group.** (B)** The heat map by expression of autophagy related genes (KEGG_REGULATION_OF_AUTOPHAGY) in tumor or normal bladder tissues of TCGA database (bladder cancer set). **(C)** Western blot analysis of autophagy-related proteins Beclin-1, LC3I, LC3II, p62 and β-Actin in T24 and UMUC3 cells with METTL16 knockdown or overexpression. **(D and E)** Representative TEM images of autophagosome (red arrow) were shown in shNC group and shMETTL16 group (Scale bars indicate 2μm,5μm). Cisplatin induced production of autophagosomes in T24 and UMUC3 cells. The number of autophagosomes increased after METTL16 knockdown.** (F and G)** Representative images with mRFP-GFP-LC3 in T24 and UMUC3 cells with or without treatment of cisplatin respectively. Yellow puncta signify autophagosomes, red arrows indicated the autophagosomes. Scale bars indicated 10 µm. Quantification of LC3 puncta were counted. Data represent the mean ± SD from five independent experiments, *P < 0.05. **(H)** CCK8 assays were used to measure the effect of CQ (25µM) on bladder cancer cells with METTL16 knockdown. CQ could partly reduce cell growth induced by knockdown of METTL16 in T24 and UMUC3 cells. Data represent the mean ± SD from three independent experiments, *P < 0.05.** (I)** Colony formation assays showed that CQ (25µM) could rescue the cell growth promoted by knockdown of METTL16 in T24 and UMUC3 cells. Data represent the mean ± SD from three independent experiments, *P < 0.05.

Obvious enrichment of autophagy pathway was found after knockdown of METTL16, following analysis of our sequencing data (Figure 5A). Combining KEGG_REGULATION_OF_AUTOPHAGY gene set with the expression of tumor or normal bladder tissues in TCGA database, we observed that key genes of autophagy were mostly overexpressed in bladder tumor tissue, which implied autophagy may play a relatively active role in bladder cancer (Figure 5B). Therefore, we speculated that METTL16 may affect the autophagy pathway of bladder cancer cells. Furthermore, we found that after knocking down METTL16, the autophagy pathway protein Beclin-1, LC3II/LC3I significantly increased, while the level of p62 remarkably decreased, meaning the promotion of autophagy. In METTL16 overexpression group, the opposite phenomenon was found (Figure 5C). TEM (transmission electron microscopy) identified that the number of autophagosomes increased after METTL16 knockdown.

After the addition of cisplatin into bladder cancer cells for 36 h, this trend can significantly increase in T24 and UMUC3 cells (Figure 5D,5E). After transfected mRFP-GFP-LC3 adenovirus reporter in T24 and UMUC3 cell which were transfected in METTL16 small interference RNA (siMETTL16) or a control (SCR-METTL16). Through verification of interference efficiency by qRT-PCR analysis and western blotting, we selected siMETTL16-2 as the siMETTL16 for subsequent experiments (Figure S5A,5B). We found that autophagy reflux was activated while interfering METTL16 and cisplatin can significantly increase this trend in T24 and UMUC3 cells (Figure 5F,5G). Chloroquine (CQ), a common autophagy inhibitor, was reported to significantly reduce the proliferation activity of bladder cancer cells^72^. Using CCK8 assay and colony formation assay, we observed that 25μM CQ inhibited the tumor cell proliferation caused by METTL16 knockdown (Figure 5H,5I). Taken together, all these results indicated that METTL16 may suppress the proliferation and cisplatin resistant of bladder cancer cells through autophagy pathway.

### PMEPA1 Interference Restored the Cell Proliferation, Apoptosis Rate and Cisplatin Resistance by Downregulating METTL16 Expression through Autophagy Pathway in Bladder Cancer

**Figure 6:**
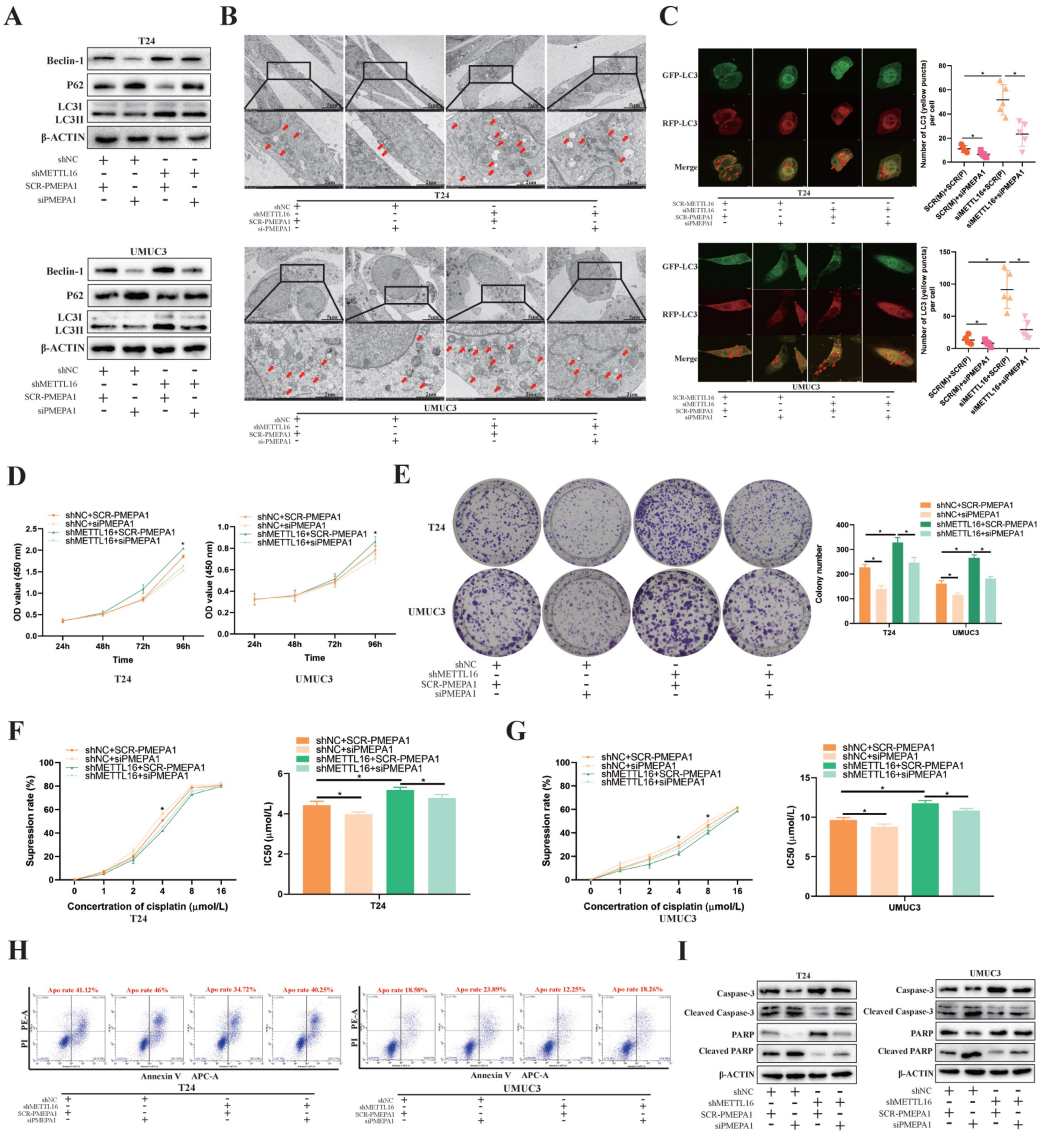
** PMEPA1 interference restored the cell proliferation, cisplatin resistance and apoptosis rate by downregulating METTL16 expression through autophagy pathway in bladder cancer. (A)** Western blot analysis of autophagy-related proteins Beclin-1, LC3I, LC3II, p62 and β-Actin in T24 and UMUC3 cells with interfering PMEPA1 after METTL16 knockdown.** (B)** After treatment of cisplatin, representative TEM images of autophagosome (red arrow) were shown (Scale bars indicate 2μm,5μm). Interference of PMEPA1 could reverse the increased autophagosomes' number caused by METTL16 knockdown.** (C)** Representative images with mRFP-GFP-LC3 in T24 and UMUC3 cells with treatment of cisplatin. Yellow puncta signify autophagosomes, red arrows indicated the autophagosomes. Scale bars indicate 10 µm. Quantification of LC3 puncta were counted. The autophagy reflux that was obviously activated by siMETTL16 was also inhibited by interference of PMEPA1. Data represent the mean ± SD from five independent experiments, *P < 0.05.** (D)** Cell proliferation assessed by CCK8 assays. Increased proliferation of bladder cancer cell lines induced by METTL16 knockdown could be reversed by interference with PMEPA1. Data represent the mean ± SD from three independent experiments, *P < 0.05. **(E)** Increased cloning number of bladder cancer cell lines induced by METTL16 knockdown could be reversed by interference with PMEPA1. Data represent the mean ± SD from three independent experiments, *P < 0.05.** (F and G)** The cisplatin inhibition rate and IC50 after treated with cisplatin for 36 hours. After the interference of PMEPA1, cisplatin inhibition rate elevated, IC50 decreased. The interference of PMEPA1 could reverse the resistance of cisplatin induced by METTL16 knockdown in T24 and UMUC3 by CCK8. Data represent the mean ± SD from three independent experiments, *P < 0.05. **(H)** The percentage of apoptotic cells after treated with cisplatin for 36 hours. The interference of PMEPA1 after METTL16 knockdown increased the apoptosis rate of bladder cancer cells induced by cisplatin. **(I)** After treated with cisplatin for 36 hours, western blot analysis of apoptosis-related proteins caspase3, PARP, Cleaved -caspase3, Cleaved-PARP and β-Actin in T24 and UMUC3 cells. The interference of PMEPA1 reversed the anti-apoptosis induced by METTL16 knockdown in T24 and UMUC3.

Based on KEGG_REGULATION_OF_AUTOPHAGY gene set, we found most autophagy related genes were positively correlated with PMEPA1 expression in TCGA-BLCA dataset (Figure S6A). To confirm that the observed phenotype was mediated by abnormal regulation of the METTL16-PMEPA1-autophagy axis, we transfected PMEPA1 small interfering RNA (siPMEPA1) or control (SCR-PMEPA1) into METTL16 knockdown and control cells for rescue experiments. Through verification of interference efficiency by qRT-PCR analysis and western blotting, we selected siPMEPA1-1 as the siPMEPA1 for subsequent experiments (Figure S7A, S7B). Western blotting results demonstrated that interfering with PMEPA1 could reverse the increased autophagy level caused by METTL16 knockdown (Figure 6A). Meanwhile, the interference of PMEPA1 could reverse the increased number of autophagosomes caused by METTL16 knockdown under electron microscope after treated with cisplatin for 36 h (Figure 6B). The autophagy reflux that obviously activated by siMETTL16 was also inhibited by interference of PMEPA1 after transfected with mRFP-GFP-LC3 adenovirus reporter (Figure 6C). Besides, CCK-8 (Figure 6D) and colony formation analysis (Figure 6E) showed that the increased proliferation of bladder cancer cell lines induced by METTL16 knockdown could be reversed by interference with PMEPA1. In addition, the interference of PMEPA1 could elevate cisplatin inhibition rate and decrease IC50, thus reversing the resistance of cisplatin induced by METTL16 knockdown in T24 (Figure 6F) and UMUC3 (Figure 6G). Furthermore, the interference of PMEPA1 increased bladder cancer cells' apoptosis rate, the level of apoptosis-related proteins Cleaved-caspase3, Cleaved-PARP expression, decreased the caspase3, PARP expression, which implied it can reverse the anti-apoptosis induced by METTL16 knockdown in T24 and UMUC3 (Figure 6H,6I). Together, knocking down PMEPA1 reversed the proliferation and cisplatin-resistance induced by METTL16 knockdown through autophagy pathway in bladder cancer cells.

### Under Hypoxia, HIF-2α Down-Regulated METTL16 Expression by Directly Binding to the METTL16 Promoter Region in Bladder Cancer Cells

**Figure 7:**
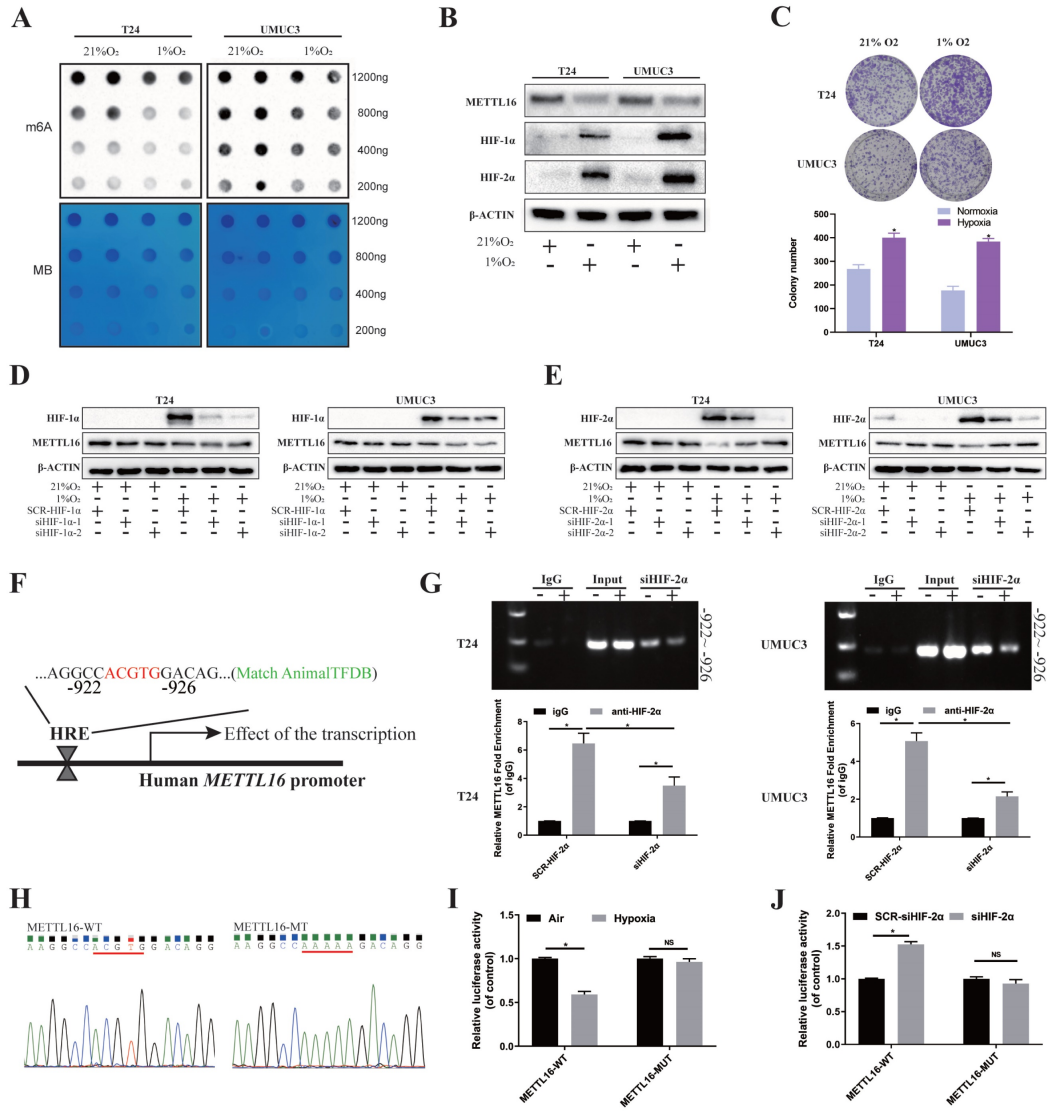
** HIF-2α down-regulated METTL16 expression by directly binding to the METTL16 promoter region in bladder cancer cells under hypoxia. (A)** M6A dot blot assays of T24 and UMUC3 cells in 21% (Normoxic) or 1% (Hypoxia) O_2_. Methylene blue (MB) stain as loading control.** (B)** HIF-1α, HIF-2α, METTL16 and β-Actin proteins expression in T24 and UMUC3 cells exposed to 1% O_2_ as compared with those exposed to 21% O_2_ for 48 hours by western blotting.** (C)** Colony formation assay in T24 and UMUC3 cells exposed to 1% O_2_ and 21% O_2_ for 7 days. Data represent the mean ± SD from three independent experiments, *P < 0.05.** (D)** HIF-1α, METTL16 and β-Actin proteins expression in T24 and UMUC3 cells exposed to 1% O_2_ and 21% O_2_ for 48 hours after interference of HIF-1α by western blotting.** (E)** HIF-2α, METTL16 and β-Actin proteins expression in T24 and UMUC3 cells exposed to 1% O_2_ and 21% O_2_ for 48 hours after interference of HIF-2α by western blotting.** (F)** The predicted HRE binding sites in the promoter region of METTL16 using AnimalTFDB database3.0.** (G)** After transduced with siHIF-2α or SCR-HIF-2α, T24 and UMUC3 cells were exposed to 1% O_2_ for 48 hours and were harvested for the ChIP assay. HIF-2α antibody was immunoprecipitated with chromatin DNA fragments, and IgG (anti-Rabbit) was applied as the negative control. The precipitated DNA was amplified by qRT-PCR. Data represent the mean ± SD from three independent experiments, *P < 0.05. **(H)** WT (ACGTG) and MT (AAAAA) luciferase reporter of the METTL16 promoter region were constructed. **(I)** Relative luciferase activity was measured in 293T cells transfected with reporter plasmids containing METTL16 promoter fragments. Then cells were exposed to 21% or 1% O2 for 48 hours. The ratio of luciferase activity was determined, the firefly luciferase activity was normalized against Renilla activity. Data represent the mean ± SD from three independent experiments, *P < 0.05.** (J)** After transduced with siHIF-2α or SCR-HIF-2α, relative luciferase activity was measured in 293T cells transfected with reporter plasmids containing METTL16 promoter fragments. Then cells were exposed to 1% O2 for 48 hours. The ratio of luciferase activity was determined, the firefly luciferase activity was normalized against Renilla activity. Data represent the mean ± SD from three independent experiments, *P < 0.05.

Rapid growth results in a hypoxic microenvironment of solid tumors, such as bladder cancer. Lots of reports demonstrated that m6A proteins and its regulation are associated with hypoxic condition. To explore the possible upstream regulatory of METTL16, we simulated the anoxic environment of bladder cancer cells by exposing T24 and UMUC3 cells in 21% (Normoxic) or 1% (Hypoxia) O_2_. Dot blot results showed that m6A level of bladder cancer cells significantly reduced in 1% O_2_ after 48 h, compared with normal oxygen condition (Figure 7A). After incubating under hypoxia condition, the protein of HIF-1α and HIF-2α were significantly increased, while protein of METTL16 was decreased in T24 and UMUC3 cells (Figure 7B). Furthermore, CCK8 and colony formation confirmed that the proliferation of T24 and UMUC3 cells under hypoxia was remarkably faster than that under normal oxygen (Figure 7C).

Then, we used small interference to knock down HIF-1α and HIF-2α respectively (Figure S8A), and found that only after interfering HIF-2α, METTL16 protein level was significantly rescued after hypoxia (Figure 7D, E). Through verification of interference efficiency by qRT-PCR analysis and western blotting, we selected siHIF-2α-2 as the siHIF-2α for subsequent experiments. Further, we found that PMEPA1 protein were also significantly upregulated in hypoxic-treated T24 and UMUC3 cells (Figure S8B). Under hypoxia, reducing METTL16 could reverse the decreased PMEPA1 protein's level caused by interfering HIF-2α (Figure S8C).

As HIFs usually regulate transcription by binding to the HRE (“ACGTG” or “GCGTG”) of the promoter region of target genes. Bioinformatics analysis (AnimalTFDB database3.0, http://bioinfo.life.hust.edu.cn/AnimalTFDB/) predicted (Figure S8D) that HIF-2α (EPAS1) may have a binding site during -922 to -926 bps in upstream of METTL16 transcription start site 2000bp near promoter region, which was consistent with the HRE (ACGTG) sequence (Figure 7F). To determine the requirement of the HRE site for HIF-2α to METTL16 promoter's transcriptional repression, we carried out the ChIP assay in T24 and UMUC3 cells to identify the binding of HIF-2α to genomic DNA. The results demonstrated that under hypoxia, compared with anti-IgG antibody, anti-HIF-2α antibody could significantly enrich METTL16 DNA with the predicted HRE sequence, while the amount of enriched DNA decreased remarkably after interfering with HIF-2α (Figure 7G). Moreover, wild (ACGTG) and missense mutant (AAAAA) luciferase reporter of the METTL16 promoter region were constructed (Figure 7H). Luciferase reporter result in 293T cell showed that the mutation of HRE almost cancelled the transcriptional repression of METTL16 promoter under hypoxic conditions (Figure 7I). While interference of HIF-2α increased the transcriptional activity of METTL16 under hypoxia, the mutation of the HER region reversed the siHIF-2α-induced transcriptional activity (Figure 7J). Together, HIF-2α down-regulated expression of METTL16 by binding to the METTL16 promoter region in bladder cancer cells under hypoxia.

## Discussion

According to current knowledge, the m6A decoration in mRNA is mainly deposited by the METTL3-METTL14 methyltransferase complex 73. Both METTL3 and METTL14 have been reported to the progression of bladder cancer via an m6A-dependent mechanism^16^, ^20^, ^21^, ^25^, ^74^. However, METTL3 and METTL14 is known to cause only about 60% of m6A modification in some type of cells 26, 28. These observations imply the existence of additional independent m6A methyltransferase. METTL16 is a recently identified m6A methyltransferase different from the METTL3/14 complex, which can independently deposit the m6A modification. In the present study, we found that the level of METTL16 was significantly downregulated in bladder cancer tissues and cell lines. And the down-expression of METTL16 was associated with TNM stage and poor prognosis of bladder cancer patients, which was consistent with Zheng *et al.* 38 reported. This indicates that METTL16 may have the potential to be a prognostic indicator for bladder tumors.

METTL16 was reported to induce initiation or progression in leukemia^31^, hepatocellular cancer^29^, pancreatic cancer^30^, breast cancer^32^, gastric cancer^33^, cervical cancer^34^ and so on^35^-^37^. Zeng *et al.*^30^ demonstrated PDAC cells with high METTL16 expression increased sensitivity to PARPi, especially when combined with gemcitabine. It indicated that METTL16 may act as an effective treatment target to suppress the resistance of drugs. In addition, METTL16 is able to accelerate the apoptosis of nucleus pulposus cells through m6A-dependent manner^75^. In the present study, we found that knockdown of METTL16 could enhance the proliferation, reduce the rate of apoptosis, and increase the resistance to cisplatin chemotherapy of T24 and UMUC3 cells *in vitro*, while overexpression of METTL16 elicited the opposite effect. Similarly, *in vivo* experiments also showed that knockdown of METTL16 in T24 cell led to the development of smaller tumors and reduce the sensitivity of bladder tumor to cisplatin chemotherapy in nude mice compared with the control group. Thus, these results confirmed that METTL16 played a suppressive role in bladder cancer, may predict the prognosis and therapeutic effect.

Primitively research of METTL16 as a methyltransferase focused on identifying specific sequences “UACAGAGAA” of few non-coding RNAs^8^, ^76^, ^77^. Recently, Su *et al.*^78^ and Han *et al.*^31^ respectively demonstrated that METTL16 could also recognize a large number of mRNA transcripts that analogous to canonical ''DRACH'' (D =A, G, or U; R = A or G; H = A, C, or U) m6A motifs which are different from the sequence found previously. While the most common modification mode of m6A methylation is binding to target gene mRNA^78^, which is consistent with the motif of our m6A-RIP-sequencing. This study confirmed that METTL16 was positively correlated with the level of m6A in bladder tumor cell line T24 and UMUC3 through dot blot. Further, RNA-seq and MERIP-seq analysis indicated that after knocking down METTL16, PMEPA1 had both m6A peak reduction and transcript level increase, suggesting that PMEPA1 may as a potential downstream target for research. Through bioinformatics analysis, western blotting, qRT-PCR and IHC assay, we confirmed that PMEPA1 was significantly negatively correlated with METTL16. High expression of PMEPA1 was associated with poor prognosis of bladder cancer patients. The methyltransferase deposits m6A within on a specific subset of cellular transcripts. M6A can be found throughout the length of transcripts, but is strongly enriched in the vicinity of stop-codons and in unusually long internal exons^73^. As m6A methyltransferases were usually found to regulate the targeted genes by affecting mRNA stability when deposits m6A in the 3'-UTR. Ni *et al.*^16^ revealed JNK signaling promotes bladder cancer immune escape by regulating METTL3-mediated PD-L1 mRNA stability. Li* et al*.^79^ reported METTL3 promotes oxaliplatin resistance of gastric cancer CD133+ stem cells by promoting PARP1 mRNA stability. Zhu *et al.*^80^ demonstrated that m6A methyltransferase KIAA1429 regulates the cisplatin sensitivity of gastric cancer cells via stabilizing FOXM1 mRNA. Liu *et al.*^81^ confirmed METTL14 regulates TIE1 mRNA stability and angiogenesis in hemorrhoids by targeting specific m6A site of the 3'-UTR. Chen *et al.*^82^ demonstrated WTAP promotes osteosarcoma tumorigenesis by repressing HMBOX1 expression in an m6A-dependent manner. Yu *et al.*^83^ found WTAP accelerates the Warburg effect of gastric cancer through regulating HK2 stability. In our study, we found it was a significantly decrease of m6A peak in the PMEPA1 mRNA 3'-UTR after knocking down METTL16 by MeRIP-seq and IGV visualization software analysis. Further research had also shown that in bladder cancer cells, METTL16 could specifically recognize the m6A sites of the 3'-UTR in PMEPA1 mRNA, which decreased the stability of PMEPA1 mRNA and eventually led to a decrease in the protein level of PMEPA1 in an m6A-dependent manner.

PMEPA1(also known as Solid Tumor-Associated 1), has been reported highly expressed in diverse type of solid tumors and implicated in tumorigenicity^84^-^92^. In addition, PMEPA1 plays an important role in promoting autophagy 93-95. In bladder cancer, Qiu *et al.*^89^ reported that PMEPA1 was high expression and involved in tumor progression and the tumor microenvironment through open database (TCGA, GEO, TIMER, and TISIDB) and several functional assays *in vitro*. This indicated that PMEPA1 may be a potential biomarker for predicting the progression and prognosis of bladder cancer. Our finding indicated that PMEPA1 and METTL16 have co-localization in bladder cancer cells. High expression of PMEPA1 led to poor prognosis. METTL16 was negatively correlated with PMEPA1 expression.

We further analyzed the sequencing data and found that there was significant enrichment in autophagy pathway after knocking down METTL16. Bioinformatics analysis results also showed that a large number of autophagy genes were highly expressed in bladder tumors. Autophagy is the major intracellular degradation system by which cytoplasmic materials are delivered to and degraded in the lysosome^55^. Many studies showed that autophagy can promote the proliferation^58^, ^65^, ^66^ and chemoresistance to cisplatin^66^, ^96^, ^97^ of bladder cancer cells. Li *et al.*^65^ reported that autophagy promotes the progression of bladder cancer cell T24 and UMUC3 by LDHA mediated metabolic reprogramming. While Lin *et al.*^72^ found chloroquin (an autophagy inhibitor) represses bladder cancer cell growth by targeting basal autophagy and enhancing apoptosis. It is consistent with the results of our study *in vitro*. In addition, it was reported that PMEPA1 co-localizes to the lysosome and late endosome^98^ and plays an important role in promoting autophagy 93-95. Luo* et al.*^93^ demonstrated that PMEPA1 could affect the chemosensitivity of lung cancer, gastric cancer and breast cancer cells through the autophagy. Through analyzing the expression correlation of bladder tumor tissues based on the TCGA database, we also found a significant positive correlation between PMEPA1 and most autophagy related genes. Therefore, we speculated that the possibility of METTL16-PMEPA1-autophagy axis. Interfering with PMEPA1 could reverse the increased autophagy level caused by METTL16 knockdown. Furthermore, interfering with PMEPA1 after knocking down METTL16 inhibited proliferation of bladder tumor cells, while increased the rate of cisplatin-induction apoptosis, the sensitivity of cells to cisplatin and the expression of apoptosis related protein. At the same time, all results confirmed that the expression of METTL16 and PMEPA1 was negatively correlated in bladder cancer tissues and cell lines, which may affect the proliferation and drug resistance of bladder tumor cells through PMEPA1 mediated autophagy, which also provided clues for the sensitization of traditional comprehensive therapy by inhibiting PMEPA1 and autophagy in bladder cancer.

Previous studies of METTL16 have focused on its structure, function^99^ and the mechanism of downstream regulation^78^, ^100^. However, the possible upstream regulation of METTL16 is also worth exploring. Hypoxia, the inadequate supply of oxygen in tissues, is an intrinsic property of the TME, which present in nearly all solid cancer sites 40. Notably, hypoxia and tumor growth form a mutually positive feedback loop. Tumor cell proliferation leads to excessive oxygen depletion and promotes a hypoxic environment in TME, which, in turn, provides conditions suitable for tumorigenesis through multiple modalities including proliferation^101^ and drug resistance^102^. Pan neerdoss* et al.*^39^ demonstrated that the level/activity of m6A writers, erasers, and readers can be regulated by hypoxia. The cellular response to hypoxia, followed by activation of HIFs family, has been reported to be emerging as an important mechanism promoting tumor chemoresistance, aggressiveness, metastasis and poor prognosis^103^. Meanwhile, as well-known transcription factors, HIFs have been reported to affect m6A level in tumor via interfering with transcription of m6A-related genes^51^-^54^. Our study found that HIF-2α was probable the upstream regulation of METTL16 in bladder cancer. Compared with normoxia, the m6A level of bladder tumor T24 and UMUC3 cells decreased under hypoxia, and the cell malignancy increased significantly. We further found that the expression of HIF-1α/ HIF-2α in T24 and UMUC3 increased after hypoxia, while METTL16 decreased significantly. By interfering of HIF-1α/ HIF-2α, we observed that it was HIF-2α instead of HIF1α, which inhibited the protein expression of METTL16 after hypoxia. According to bioinformatics by AnimalTFDB, METTL16 has promoter region that can be combined with transcription factor HIF-2α (Table S4). Then, the ChIP assay demonstrated that HIF-2α-antibody could significantly enrich METTL16 DNA with the predicted HRE sequence, whares interference with HIF-2α reduced METTL16 DNA enrichment compared with the control group after hypoxia. Furthermore, the wild-type or missense mutation luciferase reporter in HRE region of METTL16 promoter were transfected and detected in 293T cell. The results confirmed the transcription activity of this METTL16 HRE region decreased after hypoxia and restored after interfered with HIF-2α. Our results implied that under hypoxic, HIF-2α negative regulation of METTL16 through transcriptional dependent way. HIF-2α is famous in renal cancer^104^. At present, the inhibitors of HIF-2α have been put into a large number of clinical trials^105^-^107^. However, as an attractive therapeutic target, the correlation of HIF-2α with m6A related proteins has not been exploited in the bladder cancer. It is worth considering whether the inhibitor can target the combination chemotherapy to increase the sensitivity. In addition, HIFs have a significant regulatory effect on tumor immune response^108^. Whether HIF-2α inhibitor can be combined with PD-1/L1 inhibitor of immune checkpoint to sensitize the immunotherapy for bladder cancer can also be further explored. Our findings may fresh insights into the study of m6A-mediated solid tumors like bladder cancer under hypoxic microenvironment.

## Conclusions

Taken together, our results showed that METTL16 was downregulated in bladder cancer and associated with the prognosis of bladder cancer patients. Moreover, METTL16 significantly inhibited bladder cancer cell proliferation and sensitized bladder cancer cells to cisplatin *in vitro* and *in vivo* via HIF-2α-PMEPA1-autophagy axis in a m6A manner, which might provide fresh insights into bladder cancer therapy.

## Materials and Methods

### Human tissue specimens

All the bladder cancer tumors and adjacent tissue samples were gathered from patients diagnosed as bladder cancer and undergone radical cystectomy in the First Affiliated Hospital of Nanjing Medical University between 2017 and 2022. Tissue samples were frozen in liquid nitrogen or fixed by paraformaldehyde solution immediately. All patients voluntarily signed the informed consent form that the surgical tissues may be used for research purposes. The methodology used in this study was fully compliant with the guidelines of the Declaration of Helsinki and approved by the ethics committee of the First Affiliated Hospital of Nanjing Medical University (2015-SRFA-096). Follow-up data were recorded from the end of surgery to the onset of disease progression or recurrence.

### Cell culture

Seven bladder cancer cell lines (T24, RT4, UMUC3, TCC, J82, 253J and BIU87), one human ureteral epithelial immortalized cell line (SV‑HUC-1) and one mammalian HEK293T cell were obtained from the National Collection of the Authenticated Cell Cultures (Shanghai, China) and cultured in DMEM (for T24, UMUC3, TCC, J82, HEK293T)/RPMI 1640 (for RT4, BIU87)/F12K (for SV‑HUC-1) medium (Gibco®, Thermo Fisher Scientific, USA) containing 10% fetal bovine serum (Biological Industries, Israel) and 1% penicillin/streptomycin (Gibco®, Thermo Fisher Scientific, Inc. USA). All cell lines were cultured at 37°C in a humidified incubator containing 5% CO2.

Cells culture hypoxia experiment for more than 5 days was in 1% O2 and 5% CO_2_ atmosphere by hypoxic incubator (Thermo Fisher, Steri-Cycle i160, USA). While AnaeroPack (ITSUBISHI GAS CHEMICAL COMPANY, GB-C-001, JAPAN) were used for hypoxia experiments within 48 hours.

### Tissue microarray (TMA) and immunohistochemistry (IHC)

TMA was constructed from 151 cases of formalin‑fixed, paraffin‑embedded bladder cancer tissues. IHC was performed on TMA to evaluate the level of METTL16 and PMEPA1 protein expression. The specimens were rehydrated by treatment with different grades of ethanol. Microwave heating was used to isolate the antigens. After dipping in 3% H_2_O_2_ for 10 min, the slides were treated with METTL16 (1:300; Invitrogen, USA) and PMEPA1 (1:500; Proteintech, Wuhan, China) at 4°C overnight. Afterward, HRP-conjugated antibody was used to treat the slides at room temperature for 30 min. The images were captured and recorded under a microscope (Nikon Corporation, Japan). Standard staining protocols were used 49. In addition, scoring was performed for both staining intensity (SI) and the percentage of positive cells (PP) in the tissues by two urological pathologists.

### Cell transfection

Lentiviruses constructed for METTL16 knockdown or overexpression were obtained from OBIO Technology (OBIO TECHNOLOGY CORP., LTD, Shanghai, China). Cells were seeded in 6‑well plate until 30% confluence was reached, infected with METTL16 overexpression lentivirus (METTL16), a negative control (NC), METTL16 knockdown lentivirus (shMETTL16‑1 and shMETTL16‑2), and scramble control (shNC) in bladder cancer cell T24 and UMUC3. Stably transfected cells were generated by selection using puromycin (2.5 μg/ml) for 1 week.

Wild-type and mutant-type dual-luciferase reporter plasmid of METTL16, PMEPA1 and corresponding control were synthesized by GeneCopoeia (Rockville, MD, USA). PMEPA1, METTL16, HIF-1α or HIF-2α siRNA and NC plasmid (SCR) (sequences in Table S1) were obtained from Hanbio Corp (Hanbio Co. LTD, Shanghai, China). Transfections were performed using the Invitrogen^®^ Lipofectamine 3000 kit (Thermo Fisher Scientific, USA) according to the manufacturer's instructions.

### RNA isolation and quantitative reverse transcription‑ polymerase chain reaction (qRT‑PCR)

Total RNA was extracted from cell lines and tissue samples via using Invitrogen^®^ TRIzol™ reagent (Thermo Fisher Scientific, USA). Then, the RNA was reverse transcribed into cDNA using HiScript II Q RT SuperMix (Vazyme, Nanjing, China), following the manufacturer's instructions. The StepOne Plus Real-Time PCR system (Applied Biosystems; Thermo Fisher Scientific, USA) was used to perform qRT-PCR with ChamQ SYBR qPCR Master Mix (Vazyme, Nanjing, China). The primers used in qRT‑PCR were listed in Table S2.

### RNA stability

T24 and UMUC3 cells transfected with the control lentivirus, METTL16 overexpression lentivirus or METTL16 knockdown lentivirus were treated with 1.5 μg/ml Act D (Act D, Sigma-Aldrich) for 0, 2, 4, 6 and 8 h. Total RNAs were isolated, and then subjected to qRT‑PCR analysis. The level of PMEPA1 transcript was normalized to that of β-actin control.

### Western blot analysis

Cells or tissues were treated with RIPA Lysis Buffer (Beyotime, China) containing protease inhibitors (Sigma-Aldrich, USA). The concentration of proteins was measured by using a BCA Protein Assay Kit (Beyotime, Shanghai, China). Extracted proteins were separated by sodium dodecylsulfate-polyacrylamide gel electrophoresis and transferred onto a polyvinylidene fluoride (Millipore, USA). The membrane was blocked with 5% skim milk powder and incubated with primary antibodies for METTL16 (1:500, Invitrogen, USA), PMEPA1 (1:1000, Proteintech, China), caspase-3 (1:1000, CST, USA), Cleaved caspase-3 (1:1000, CST, USA), PARP (1:1000, CST, USA), Cleaved PARP (1:1000, CST, USA), HIF-2α (1:1000, Abcam, USA), HIF1α (1:1000, CST, USA), Beclin-1(1:1000, CST, USA), P62 (1:1000, CST, USA) or LC3A/B (1:1000, CST, USA) at 4°C overnight. After washing three times in TBS containing Tween-20 (TBST), the membranes were incubated with secondary antibody (1:5000, CST, USA) at room temperature for 2 h. Band signals were detected using a chemiluminescence system (Bio‑Rad Laboratories, USA) and analyzed using Image Lab Software. The protein levels were normalized to β-actin (1:1,000; CST, USA).

### Cell proliferation and colony formation assays

Pretreated cells were counted and seeded into a 96‑well plate at a density of 1.5×10^3^ (T24) cells/well, or 2×10^3^ (UMUC3) cells/well. Cell proliferation was measured after 24, 48, 72, and 96 h using the cell counting kit‑8 (CCK‑8) assay (Vazyme, Nanjing, China). The absorbance was measured at 450 nm with a microplate reader following incubation at 37°C for 1 h according to the manufacturer's instructions.

For the colony formation assay, pretreated cells were seeded into 6‑well plates (800 cells/well). The cells were incubated for 7-14 days. The colonies were fixed with 4% paraformaldehyde and washed with PBS. After stained with crystal violet, the visible colonies were counted.

For the inhibitor experiments, Chloroquine-CQ (MedChemExpress, Shanghai, China) was added into the wells at the concentration of 25 μM after cell attachment^72^, ^109^.

### Cell cycle assay

Tumor cells (5×10^5^) were collected, and then fixed with 75% pre‑cold ethanol for 24 h at ‑20°C. Cells were assessed by flow cytometry (Becton, Dickinson and Company, USA) after staining with propidium iodide (BD Biosciences, USA). The cell cycle was analyzed via Cell Quest Modfit software version 5.0.

### IC_50_ determination

The transfected cells were seeded into a 96‑well plate at a density of 3000 cells per well and incubated overnight in an incubator. Three parallel wells were set up. Subsequently, the transfected cells were treated with 64, 32, 16, 8, 4, 2 ,1 or 0 µM cisplatin (TCI, Japan) for 24 h. Cell viability was measured by the CCK‑8(Vazyme, Nanjing, China) method according to the manufacturer's instructions. IC_50_ values were calculated by software SPSS. Suppression rate of cisplatin was 1- (OD value of x µM/ OD value of 0 µM) *100%.

### Apoptosis assay

The tumor cells were treated with cisplatin (TCI, Tokyo, Japan) for 48 h, and the cell apoptosis was detected by Annexin V‑APC and PI (Fcmacs Company, Nanjing, China), according to manufacturer's instructions. Then, the cells were analyzed with flow cytometry (Becton, Dickinson and Company, USA).

### Autophagic flux analysis

T24 and UMUC3 cells were transfected with mRFP-GFP-LC3 adenovirus (KEYGEN BIOTECH CORP., LTD, China) according to the manufacturer's instructions. Twenty-four hours after transfection, cells were cultured for 24 h with or without cisplatin (TCI okyo, Japan). Then, cells were scanned with a confocal microscope (FV10i, Olympus, Japan). 5 cells in each group were counted. Yellow spots were merged by RFP and GFP puncta, indicated autophagosomes.

### Transmission electron microscopy

T24 and UMNUC3 cells were fixed with ice-cold glutaraldehyde (Servicebio, Wuhan, China) and further processed by the Core Facility (Servicebio, Wuhan, China). The specimens were observed with a JEOL JEM-2100 transmission electron microscope.

### Xenograft experiments* in vivo*

Animal studies were approved by the Animal Research Ethics Committee of Nanjing Medical University. The methodology used in this study was approved by the ethics committee of the Nanjing Medical University (IACUC-2101028). The BALB/c nude mice (4 weeks old) were obtained from the Model Animal Research Center of Nanjing University. T24 cells stably transfected with the METTL16 knockdown lentivirus (shMETTL16) or control lentivirus (shNC) were injected subcutaneously into the axilla of each mouse. Mice in the cisplatin group were injected intraperitoneally with cisplatin (3 mg/kg body weight, three times every week) starting from day 7 of tumor inoculation^110^, ^111^. Mice in the control group were injected intraperitoneally with equal volume of saline at the same time. Tumor growth was monitored every 3 days by measuring the width (W) and length (L) with calipers, and the volume (V) of the tumor was calculated using the formula V= (length x width^2^)/2. At 2 weeks after injection of cisplatin, the mice were euthanized and tumors were removed, weighed, fixed and embedded for IHC.

### M6A dot blots assay

The extracted mRNA using for m6A dot blotting was measured using NanoDrop Nd-1000 spectrophotometer (Agilent, Santa Clara, USA). In a Bio‑Dot apparatus (Bio‑Rad Laboratories, Inc. USA), the poly(A)^+^ RNAs were denatured by heating at 65°C for 5 min, then transferred onto a nitrocellulose membrane (Amersham; GE Healthcare, USA). The membranes were UV cross-linked, then blocked, and incubated with m6A antibody (1:1000; Abcam, USA) overnight at 4°C, followed by incubation with IgG (1:4000; CST, USA). Chemiluminescence system (Bio‑Rad Laboratories, USA) was used for visualizing the membranes. To ensure consistency, the membrane stained with 0.02% methylene blue was used as a loading control.

### Dual‑luciferase reporter assay

T24, UMUC3 or 293T cells were co-transfected with plasmids containing 3′-UTR of wild-type or mutant fragments (5′-GGAC-3′ deletion mutation) from PMEPA1 and plasmids with wild-type METTL16 or mutant METTL16 (mutant HRE: 5′-ACGTG-3′ to 5′-AAAAA-3′) by using Lipofectamine 3000. After incubation for 48 hours, the luciferase activity was determined by using the dual-luciferase reporter assay system (Promega, USA). Finally, the relative luciferase activity was normalized to the renilla. Each experiment was repeated thrice.

### RNA immunoprecipitation assay (RIP)

RIP assay was performed using the Magna RIP™ RNA‑Binding Protein Immunoprecipitation kit (MilliporeSigma, USA) according to the manufacturer's instructions. Briefly, T24 and UMUC3 cells were lysed with RIPA lysis buffer. Cell lysates were immunoprecipitated with anti METTL16 antibody or IgG (anti- Rabbit) at 4°C overnight. After purification of the RNA, qRT‑PCR was performed to measure the levels of PMEPA1 transcript in the METTL16 or IgG immunocomplexes.

### M6A RNA immunoprecipitation (MeRIP)-qRT-PCR assay and MeRIP Sequencing (MeRIP-seq)

For the m6A RNA binding experiments or MeRIP-seq, the RNAs of bladder cancer cells stably transfected with either the lentiviral knock-down or control METTL16 vector were collected. RNAs were treated by Fragmentation Reagents (Invitrogen, USA) for 5min on 70℃. Immunoprecipitations were performed using an anti‑m6A antibody (1:1,000, Abcam, USA) previously bound to magnetic Life Technologies^®^ Dynabeads (Thermo Fisher Scientific, Inc USA) in the IP buffer (Magna RIP™ RNA‑Binding Protein Immunoprecipitation kit; Millipore Sigma, USA) and incubated with DNA‑free fragmented RNAs. After incubating with elution buffer (Sigma-Aldrich) for 1h at 4℃, RNAs were eluted from the beads. After precipitation, input and eluted RNAs were obtained, subsequently reverse transcribed into cDNA and subjected to qRT-PCR for PMEPA1 and normalized to input. The primers used for MeRIP-qRT‑PCR were listed in Table S2

The products were sequenced and analyzed by RiboBio Co., Ltd. (Ribobio, China) for MeRIP-seq. Effective reads from the input sample were used for RNAseq analysis and the read count value of each transcript was calculated using HTSeq (version 0.6.0). Differentially expressed genes were identified using the DEseq/DESeq2/edgeR/DEGseq R package according to the following criteria: |log2 (fold change) |≥1.

### Chromatin immunoprecipitation (ChIP) assay

ChIP assay was performed using the CHIP-IT Express kit (53008, Active Motif, USA) following the manufacturer's protocol. DNA was sheared into fragments between 300- 1000 bp by a sonicator (SONIC, VCX-130) on the ice at 25% amplitude (20 s pulse followed by 30 s pause for 10 cycles). Subsequently, equal samples quantified by DNA concentration were incubated with HIF-2α/EPAS1 antibody (Novusbio, USA) or IgG-negative control with 30 μL magnetic protein G beads overnight at 4°C with rotation. After decrosslinking and proteinase treatment, the purified DNA was collected and subjected to standard PCR techniques. The primers are included in supplementary Table S3.

### Bioinformatic analysis

Expression from UALCAN survival analysis and the IHC images from The Human Protein Atlas (HPA) database. For other bioinformatic analysis, we downloaded the clinical data for METTL16 and PMEPA1 from the TCGA dataset. Predicted the chemotherapeutic response for each sample based on the Genomics of Drug Sensitivity in Cancer (GDSC) pharmacogenomics database (https://www.cancerrxgene.org/). The prediction process was implemented by R package “pRRophetic”. The half-maximal inhibitory concentration (IC50) was estimated by ridge regression. All parameters were set as the default values. Using the batch effect of combat and tissue type of all tissues, and the duplicate gene expression was summarized as mean value. All the above analysis methods and R package were implemented by R foundation for statistical computing (2020) version 4.0.

### Statistical analysis

SPSS 19.0 software (IBM, Chicago, IL, USA) was used for statistical tests. Measurement data were tested by Student's t test and count data were tested by the χ2 test or Fischer exact test. Survival was calculated from the date of diagnosis. Kaplan-Meier curves and log-rank test for significance were used for survival analysis. Univariate and multivariate analyses were conducted with Cox proportional hazards regression model. Statistical correlation was analyzed by Pearson's correlation coefficient analysis. All data are presented as the mean ± standard deviation (SD) from three different independent experiments. P < 0.05 was considered significant.

## Supplementary Material

Supplementary figures and tables.

## Figures and Tables

**Table 1:** Association of METTL16 expression with clinicopathologic characteristics of the bladder cancer patients

Parameters	Number of cases	METTL16 expression		P -value
		Low (n=122)	High (n=29)	
Age (years)				0.531
<60	35	27	8	
≥60	116	95	21	
Gender				0.920
Male	124	100	24	
Female	27	22	5	
Histological grade				0.978
Low	18	14	4	
High	133	108	25	
TNM stage				0.017*
Ta-T1	69	50	19	
T2-T4	82	72	10	
PMEPA1				0.002*
Negative	39	25	14	
Positive	112	97	15	

*Statistically significant.

## Abbreviations

m6A: N6-methyladenosine
METTL16: Methyltransferase-like 16
qRT-PCR: Quantitative real-time PCR
RIP: RNA immunoprecipitation
MeRIP: methylated RNA immunoprecipitation
ChIP: Chromatin immunoprecipitation
IHC: Immunohistochemistry
PMEPA1: prostate transmembrane protein androgen induced-1
HIFs: hypoxia-inducible factors
HREs: hypoxia response elements
IGV: Itegrative Genomics Viewer
