# Supplementary material for: METTL16 suppressed the proliferation and cisplatin-chemoresistance of bladder cancer by degrading PMEPA1 mRNA in a m6A manner through autophagy pathway
Source: Int J Biol Sci. 2024 Feb 4;20(4):1471–91. doi: 10.7150/ijbs.86719 (PMC10878153; doi:10.7150/ijbs.86719)
Supplement: Supplementary file 1 — Supplementary figures and tables. [file ijbsv20p1471s1.pdf]

Supplemental material

Supplemental Figure S1

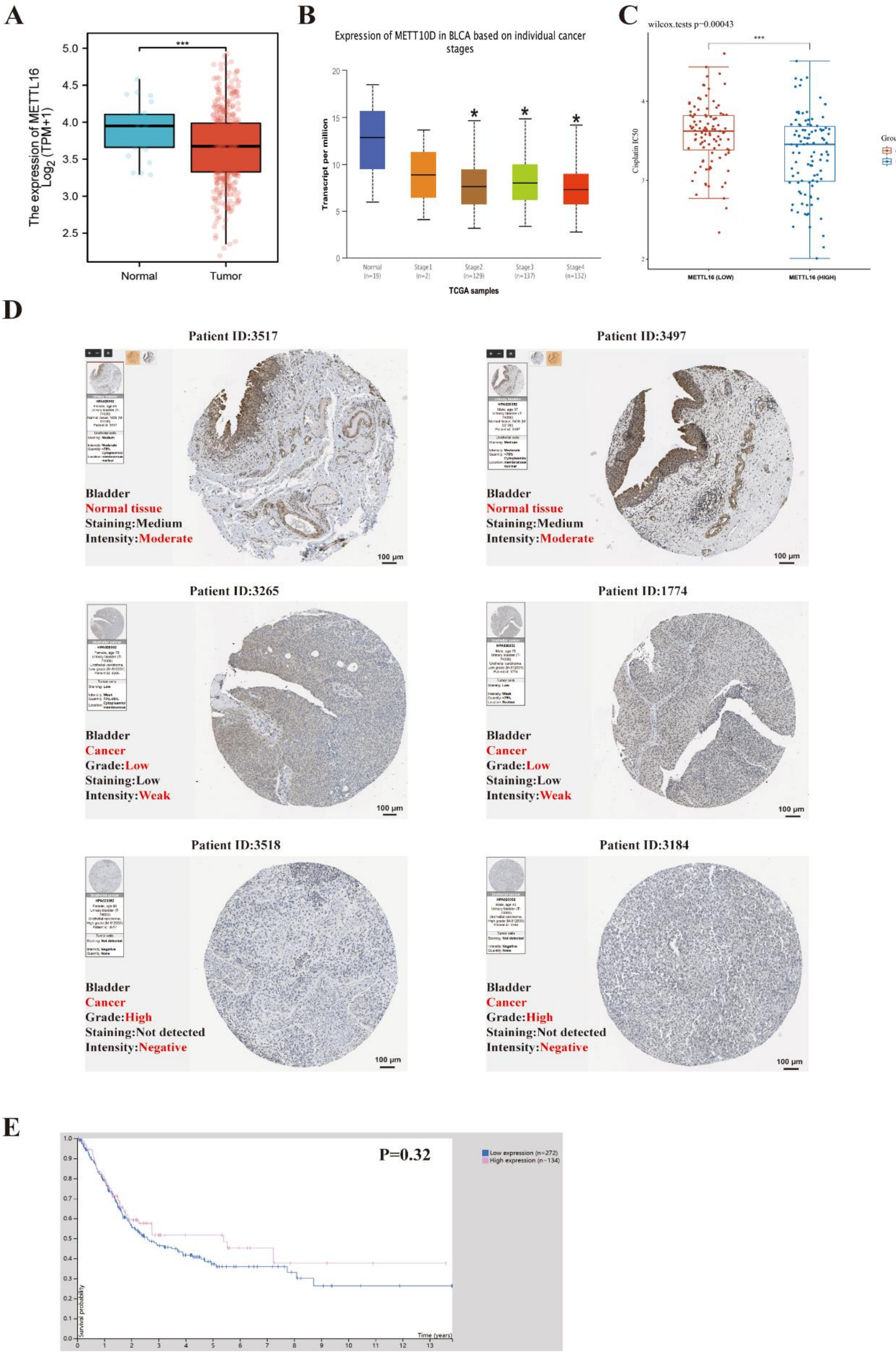

METTL16 was downregulated in bladder cancer tissues and associated with patient prognosis based on TCGA and HPA database.

(A) METTL16 expression of bladder cancer and normal bladder tissues in TCGA database. ***P < 0.001 **(B)** Expression of METTL16 (METT10D) in bladder cancer on individual cancer stages in TCGA database by UALCAN. *P < 0.05 **(C)** METTL16 expression is negatively correlated with cisplatin IC50 in bladder tumors in TCGA and Drug Sensitivity in Cancer (GDSC) database. P = 0.00043. **(D)** Representative images with the IHC of METTL16 in low- or high-grade bladder cancer from HPA database. The staining of METTL16 was relative obviously in bladder normal tissue. While the staining of METTL16 was weak or not detected in bladder cancer. Scale bars indicated 100μm. **(E)** Kaplan-Meier survival curves of overall survival in 406 bladder cancer patients based on METTL16 by HPA database. Low expression METTL16 patients had poor survival. The log-rank test was used to compare differences between two groups (P = 0.32).

Supplemental Figure S2

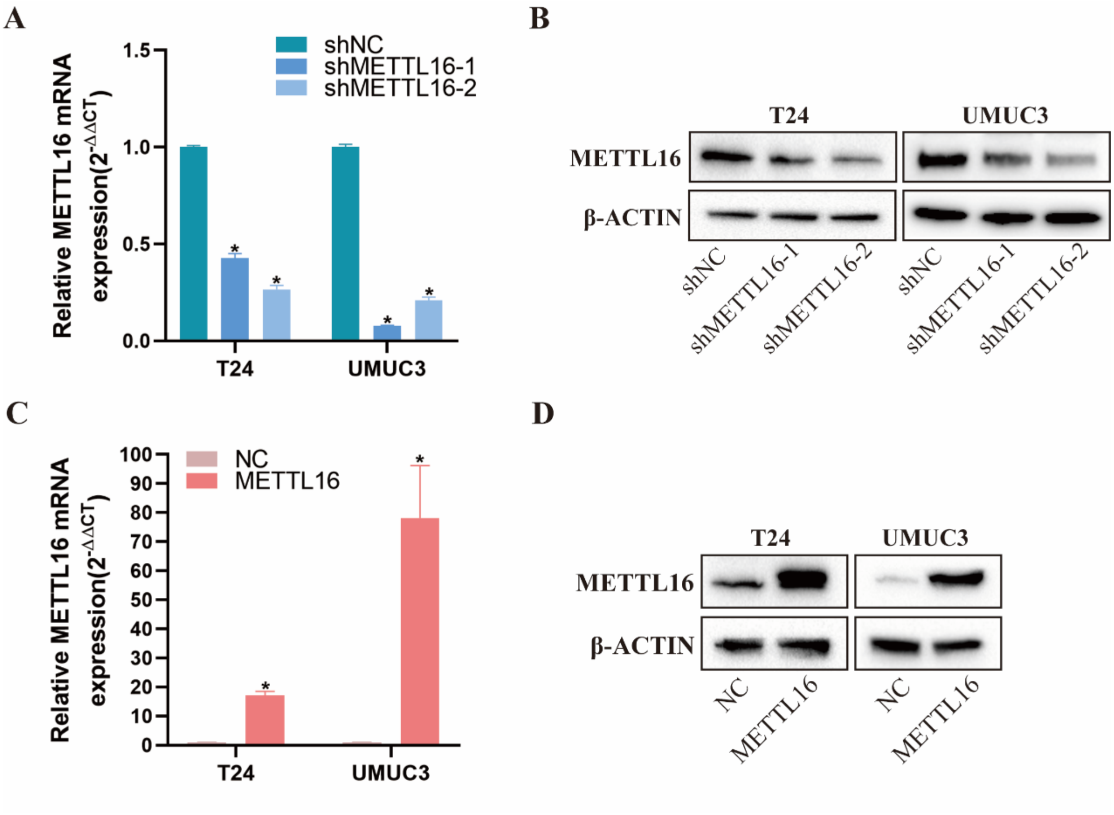

The efficiency of METTL16 knockdown and overexpression in bladder cancer cell lines.

(A, B, C, D) Validation of the overexpression and knockdown efficacy of METTL16 in T24 and UMUC3 cell lines by qRT-PCR and western blot. Data represent the mean ±SD from three independent experiments. *$P<0.05$

Supplemental Figure S3

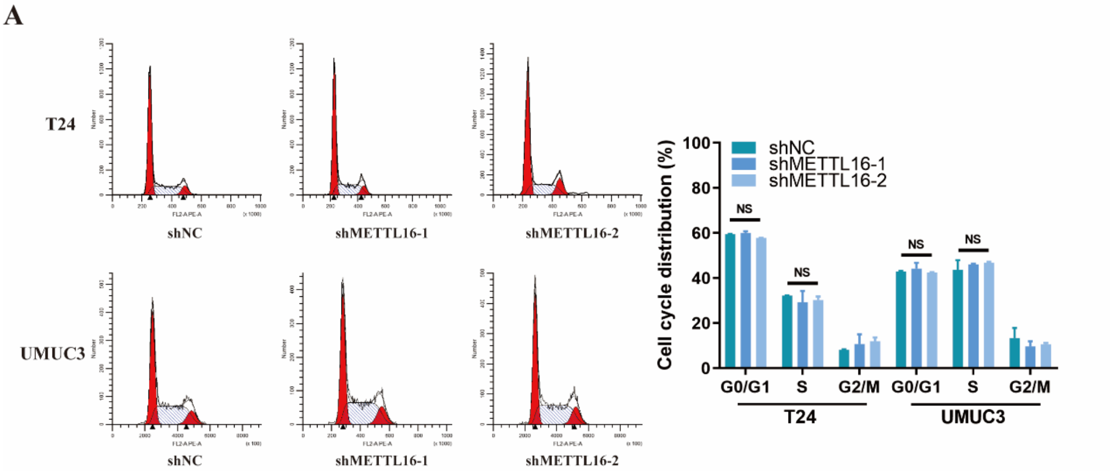

Cell cycle assay of METTL16 knockdown in bladder cancer cell lines.

(A) Cell cycle analyzed by flow cytometry. Histogram indicated the percentage of cells in G0/G1, S and G2/M. Data represent the mean ± SD from three independent experiments. NS: $P > 0.05$

Supplemental Figure S4

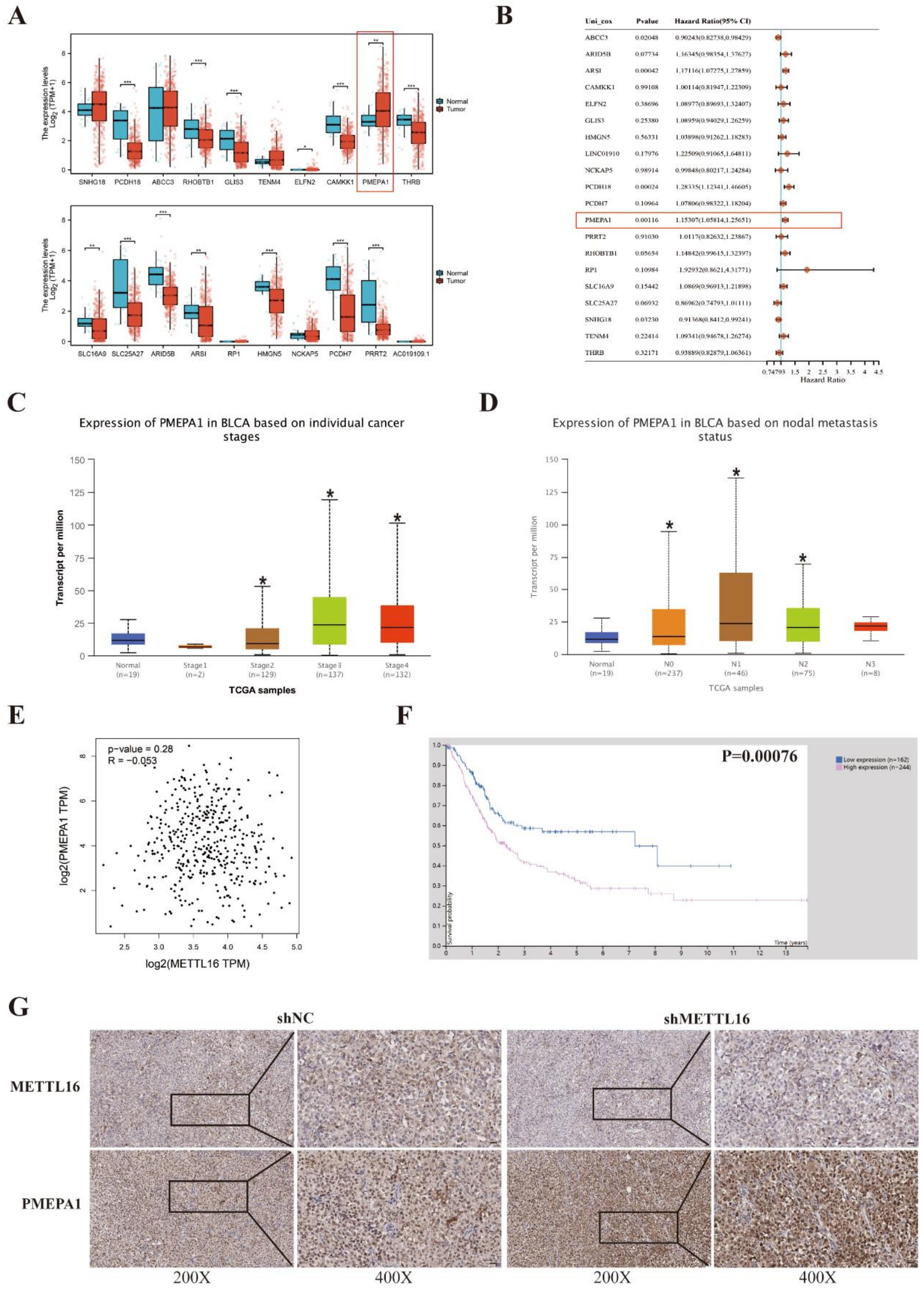

Supplementary materials of PMEPA1 as a downstream target gene for METTL16 in bladder cancer by bioinformatics and IHC.

(**A**) Top 20 candidate downstream genes (SLC16A9, SLC25A27, ARID5B, ARSI, RP1,

HMGN5, NCKAP5, PCDH7, PRRT2, LINC01910, SNHG18, PCDH18, ABCC3, RHOBTB1, GLIS3, TENM4, ELFN2, CAMKK1, PMEPA1, THRB) expression of bladder cancer and normal bladder tissues in TCGA database. *$P < 0.05$, **$P < 0.01$, ***$P < 0.001$. **(B)** PMEPA1 expression was associated with poor OS in bladder cancer (HR=1.15307, P=0.00116). **(C)** Expression of PMEPA1 in bladder cancer on individual cancer stages in TCGA database by UALCAN. *$P < 0.05$. **(D)** Expression of PMEPA1 in bladder cancer on nodal metastasis status in TCGA database by UALCAN. *$P < 0.05$. **(E)** A negative correlation trend between the expression of PMEPA1 and METTL16 from TCGA database. R=-0.053, P=0.28. **(F)** Kaplan-Meier survival curves of overall survival in 406 bladder cancer patients based on PMEPA1 by HPA database. High expression PMEPA1 patients had poor survival. The log-rank test was used to compare differences between two groups (P = 0.00076). **(G)** IHC analysis from subcutaneous xenograft tumor model showed that the expression of PMEPA1 increased after METTL16 knockdown at 200X (left) or 400X (right) magnification. Scale bars indicated 50 μm (200X), 20 μm (400X).

Supplemental Figure S5

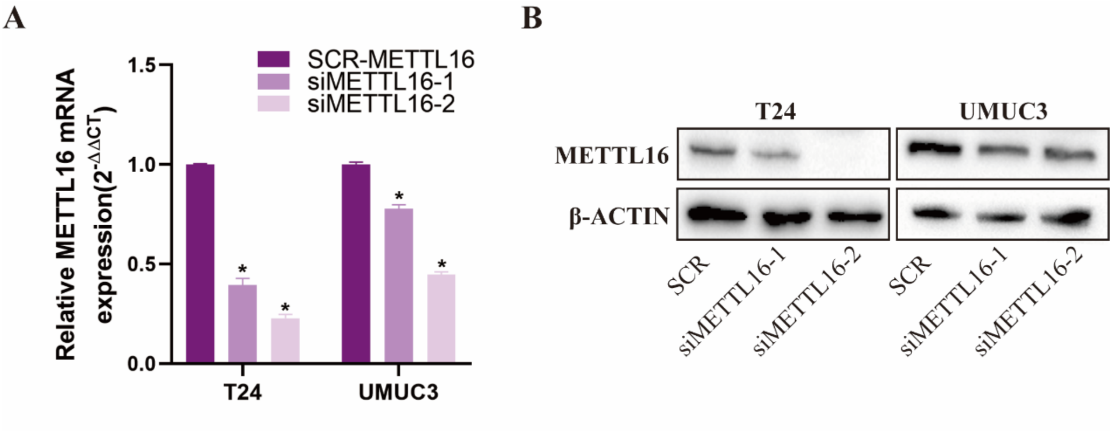

The efficiency of METTL16 small interference or a control in bladder cancer cell lines.

(A,B) Validation of the siMETTL16 and SCR-METTL16 efficacy of METTL16 in T24 and UMUC3 cell lines by qRT-PCR and western blot. Based on the above results, we selected siMETTL16-2 as the siMETTL16 for subsequent experiments. Data represent the mean ±SD from three independent experiments. *P<0.05

Supplemental Figure S6

A

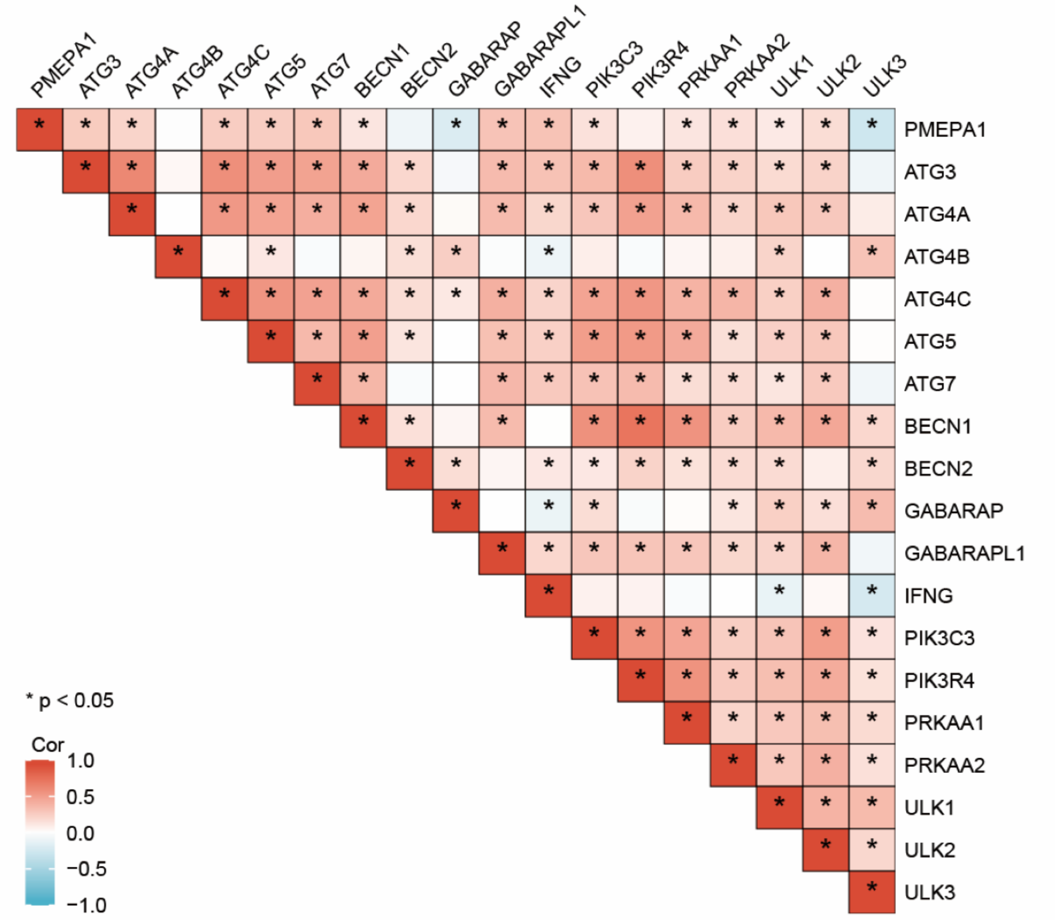

Autophagy related genes were positively correlated with PMEPA1 expression in bladder cancer.

(A) Heat map of the specific associations of the autophagy related genes from KEGG_REGULATION_OF_AUTOPHAGY correlated with PMEPA1 in bladder cancer.

Supplemental Figure S7

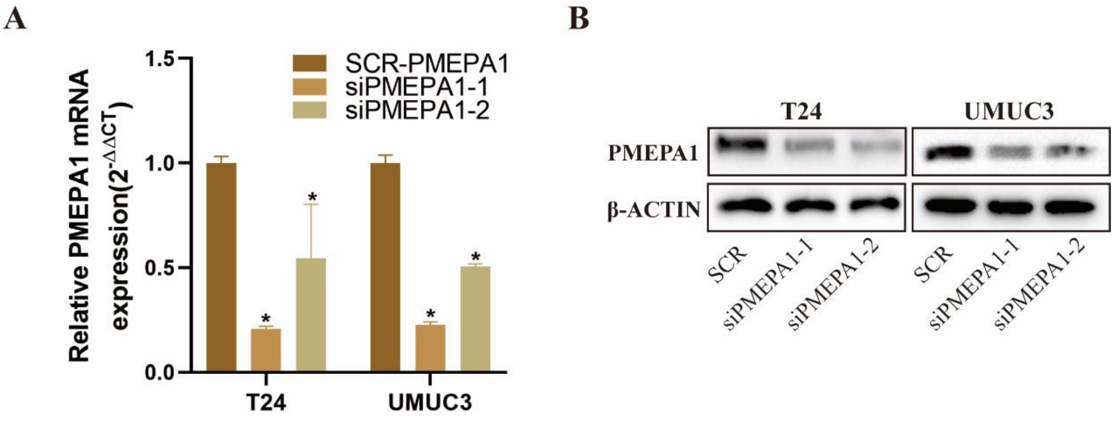

The efficiency of PMEPA1 small interference or a control in bladder cancer cell lines.

(A,B) Validation of the siPMEPA1 and SCR-PMEPA1 efficacy of PMEPA1 in T24 and UMUC3 cell lines by qRT-PCR and western blot. Based on the above results, we selected siPMEPA1-1 as the siPEMAP1 for subsequent experiments. Data represent the mean ±SD from three independent experiments. *P<0.05

Supplemental Figure S8

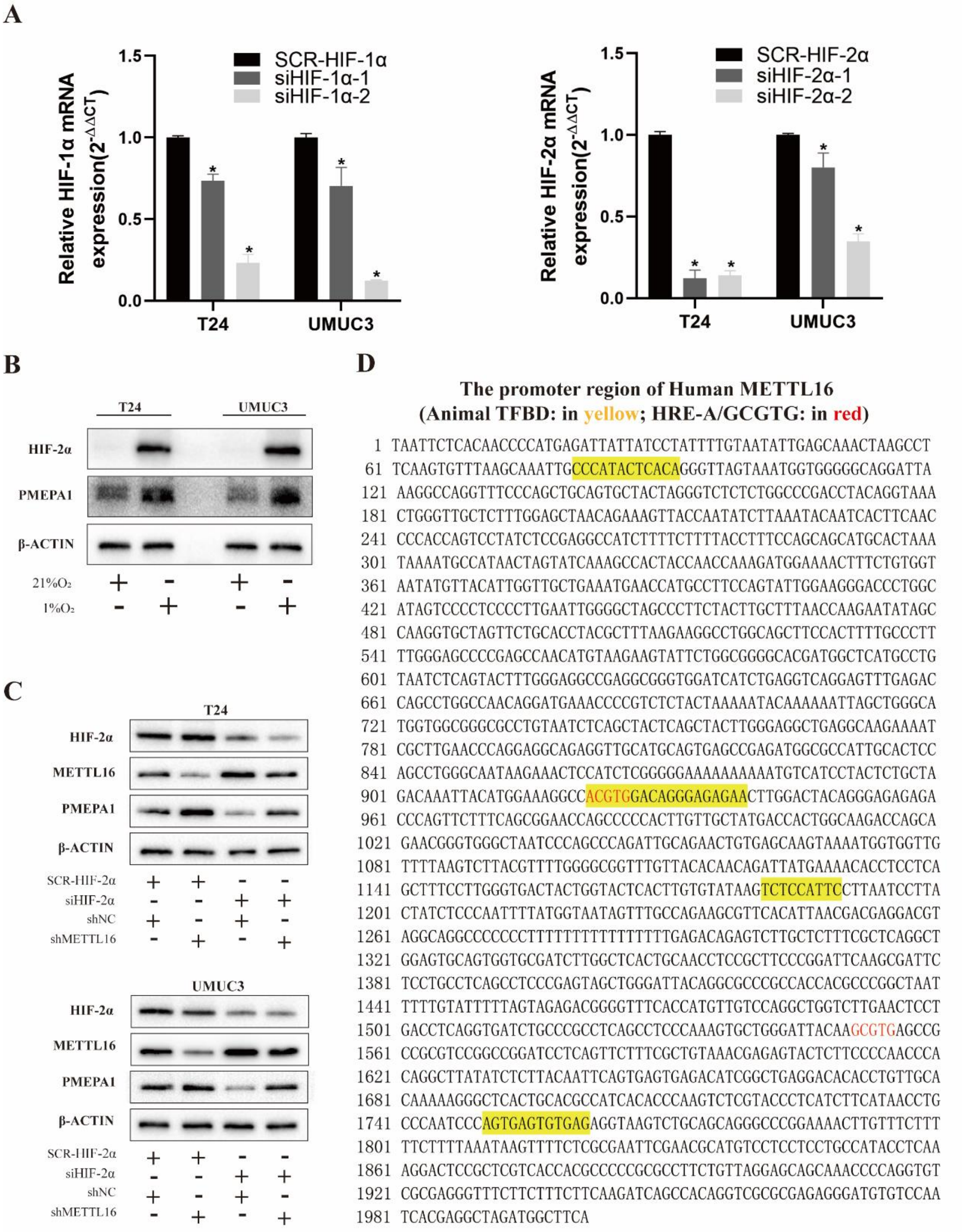

The efficiency of HIF-1α, HIF-2α small interference or a control in bladder cancer cell lines.

(A) Validation of the siHIF-1α, siHIF-2α and SCR-HIF-1α, SCR-HIF-2α efficacy of HIF-1α or HIF-2α in T24 and UMUC3 cell lines by qRT-PCR. Based on the above results, we selected siHIF-2α-2 as the siHIF-2α for subsequent experiments. Data

represent the mean ±SD from three independent experiments. *$P<0.05$. **(B)** HIF-2α, PMEPA1 and β-Actin proteins expression in T24 and UMUC3 cells exposed to 21% O_2 and 1% O_2 for 48 hours by western blotting. **(C)** Western blot analysis of HIF-2α, METTL16,PMEPA1 and β-Actin proteins expression in T24 and UMUC3 cells exposed to 1% O_2 for 48 hours with knocking down METTL16 after interfering HIF-2α. **(D)**Schematic of the promoter region of human METTL16. The binding sites of HIF-2α (EPAS1) on METTL16 predicted by TFBD database were labeled in yellow and the HRE sequence was labeled in red.

Supplemental Tables

Table S1. Small interference sequences used in Cell transfection.

Name	Species		Sequences (5′-3′)
Si-METTL16	Homo	Forward	5′- GGAUGCUCUUAAAGAAGAAdTdT -3′.
		Reverse	5′- UUCUUCUUUAAGAGCAUCCdTdT -3′
Si-PMEPA1-1	Homo	Forward	5′- GAGUUUGUUCAGAUCAUCAUCdTdT -3′
		Reverse	5′- GAUGAUGAUCUGAACAAACUCdTdT -3′
Si-PMEPA1-2	Homo	Forward	5′- GUCCCUAUGAAUUGUACGUUUdTdT -3′
		Reverse	5′- AAACGUACAAUUCAUAGGGACdTdT -3′
Si-HIF-1α-1	Homo	Forward	5′- GAAGAACUAUGAACAUAAAdTdT -3′
		Reverse	5′- UUUAUGUUCAUAGUUCUUCdTdT -3′
Si-HIF-1α-2	Homo	Forward	5′- GAAUUGGAAGAAAUCAGAAdTdT -3′
		Reverse	5′- UUCUGAUUUCUUCCAAUUCdTdT -3′
Si-HIF-2α-1	Homo	Forward	5′- GGACAACUUGUACCUGAAAdTdT -3′
		Reverse	5′- UUUCAGGUACAAGUUGUCCdTdT -3′
Si-HIF-2α-2	Homo	Forward	5′- AGGUGAAAGUCUACAACAAdTdT -3′
		Reverse	5′- UUGUUGUAGACUUUCACCUdTdT -3′

Table S2. Oligonucleotide sequences used in this study.

Primes	Species		Sequences (5′-3′)
METTL16	Homo	Forward	5′- AGGGAGTAAACTCACGAAATCCT-3′.
		Reverse	5′- AACCCCTTGTATGCGAAGCTC-3′
PMEPA1	Homo	Forward	5′- TGTCAGGCAACGGAATCCC-3′
		Reverse	5′- CAGGTACGGATAGGTGGGC-3′
HIF-1α	Homo	Forward	5′- GAACGTCGAAAAGAAAAGTCTCG-3′
		Reverse	5′- CCTTATCAAGATGCGAACTCACA-3′
HIF-2α	Homo	Forward	5′- CGGAGGTGTTCTATGAGCTGG-3′
		Reverse	5′- AGCTTGTGTGTTCGCAGGAA-3′
β-actin	Homo	Forward	5′- AGCGAGCATCCCCCAAAGTT-3′
		Reverse	5′- GGGCACGAAGGCTCATCATT-3′
PMEPA1 (MeRIP assay)	Homo	Forward	5′-GTGACCAGAGGAGAGCAT -3′.
		Reverse	5′- TTAGCTACTAGTCAGCCAT-3′

Table S3. Primer used in ChIP assay.

Antibody	Location		Sequence (5′to 3′)
METTL16	798 to 1014	Forward	5′-CAGAGGTTGCATGCAGTGAG-3′
		Reverse	5′-TCTTGCCAGTGGTCATAGCA-3′

Table S4. The putative binding sequences of HIF-2α in the METTL16 promoter region.

Name	Score	Start	End	Strand	Predicted sequence
HIF-2α	12.4079	1750	1761	-	CTCACACTCACT
HIF-2α	8.98182	924	939	+	GTGGACAGGGAGAGAA
HIF-2α	10.6842	1181	1189	+	TCTCCATTC
HIF-2α	10.7632	81	92	+	CCCATACTCACA
HIF-2α	7.76364	827	842	-	CTGGAGTGCAATGGCG

Table S5: Association of PMEPA1 expression with clinicopathologic characteristics of the bladder cancer patients

Parameters	Number of cases	PMEPA1 expression		P -value
		Low (n=39)	High (n=112)	
Age (years)				0.388
<60	35	11	24	
≥60	116	28	88	
Gender				0.142
Male	124	29	95	
Female	27	10	17	
Histological grade				0.102
Low	18	8	10	
High	133	31	102	
TNM stage				**0.007***
Ta-T1	69	25	44	
T2-T4	82	14	68	

*Statistically significant.
